# Targeting the amino acid metabolic axis: the Achilles’ heel of tumor cells

**DOI:** 10.1007/s00726-026-03522-4

**Published:** 2026-04-17

**Authors:** Zheyu Hu, Yiwei Wang, Qian Ma

**Affiliations:** Department of Obstetrics and Gynecology, First Affiliated Hospital of Zhengzhou University, Zhengzhou, 450052 China

**Keywords:** Amino acid metabolic reprogramming, Metabolic addiction, Immunosuppression, Combination therapy, Tumor microenvironment

## Abstract

Cancer is characterized by profound reprogramming of its metabolic programs, with the unending demand for exogenous amino acids by tumor cells serving as a hallmark manifestation. While this high dependency supports rapid proliferation, it exposes a critical vulnerability: disruption of amino acid supply can specifically trigger metabolic catastrophe in cancer cells. Furthermore, tumor cells exploit this metabolic reprogramming to deplete key amino acids in the microenvironment, thereby suppressing T-cell function and facilitating immune evasion. This review systematically elucidates therapeutic strategies targeting four critical amino acid metabolic axes (glutamine, arginine, tryptophan, and methionine). We delve into how inhibition of glutamine metabolism disrupts tumor bioenergetics, how arginine deprivation selectively targets cells with synthetic defects, and how methionine restriction interferes with key epigenetic regulation. Additionally, we explore interventions for these four amino acid metabolic axes to reverse immunosuppression. Convincing preclinical and clinical evidence demonstrates that these strategies, whether as monotherapy or rational combinations with conventional treatments, exhibit significant antitumor efficacy and substantial clinical translation potential. By integrating metabolic and immunological perspectives and critically assessing translational challenges, this review aims to provide a roadmap for future development of precision combination strategies capable of overcoming drug resistance and reshaping the immune microenvironment.

## Introduction

The initiation and progression of tumors are driven not only by genetic mutations but also accompanied by profound metabolic remodeling to meet the massive demands of malignant proliferation. Beyond the classical Warburg effect, cancer cells exhibit abnormally active uptake and metabolic utilization of amino acids, which serve not only as the cornerstone of biosynthesis but also as the core for maintaining energy homeostasis, redox balance, and the activity of key signaling pathways (e.g., mTOR) (Pavlova and Thompson 2016). However, this excessive dependence of cancer cells on specific exogenous amino acids—referred to as ‘metabolic addiction’—not only serves as a hallmark distinguishing them from normal cells but also constitutes their fatal metabolic vulnerability (‘Achilles’ heel’). The underlying mechanism lies in the epigenetic silencing or downregulation of key synthetic enzymes (e.g., ASS1, glutamine synthetase) during malignant transformation in many cancer cells, leading to impaired de novosynthesis of non-essential amino acids and resulting in a “nutritional deficiency” state (Huang et al. 2013; Guo et al. 2021). Therefore, this unique metabolic defect in cancer cells provides an ideal window for implementing precision-targeted therapy based on the principle of ‘synthetic lethality’: selective blockade of amino acid supply through dietary intervention, enzymatic depletion, or small molecule inhibition can theoretically achieve precise eradication of cancer cells with minimal impact on metabolically intact normal tissues (Vettore et al. 2020).

### Metabolic addiction: the achilles’ heel of tumor cells

The metabolic reprogramming of cancer cells often leads to an “addiction” to specific exogenous amino acids, such as glutamine, arginine, tryptophan, and methionine, which are indispensable for maintaining biosynthesis, energy homeostasis, and redox balance. This dependency exposes a critical vulnerability of cancer cells—interrupting the supply of amino acids can induce metabolic crises and selective cell death (Wang and Wan 2022).

The feasibility of targeting amino acid metabolism has been preliminarily validated in clinical explorations for solid tumors. Among these, arginine deprivation therapy serves as a representative example. In various malignant tumors (e.g., hepatocellular carcinoma, malignant pleural mesothelioma), downregulation or silencing of Argininosuccinate synthase 1 (ASS1) leads to the inability of cancer cells to synthesize arginine, resulting in “arginine nutritional deficiency” (Dillon et al. 2004). Leveraging this vulnerability, polyethylene glycol-arginine deiminase (ADI-PEG20) can selectively induce metabolic crisis and cell death in ASS1-deficient tumor cells by depleting arginine in the bloodstream (Rogers et al. 2021). These successful cases demonstrate that targeting the metabolism and supply of amino acids has become a promising strategy for cancer treatment.

**Fig. 1:**
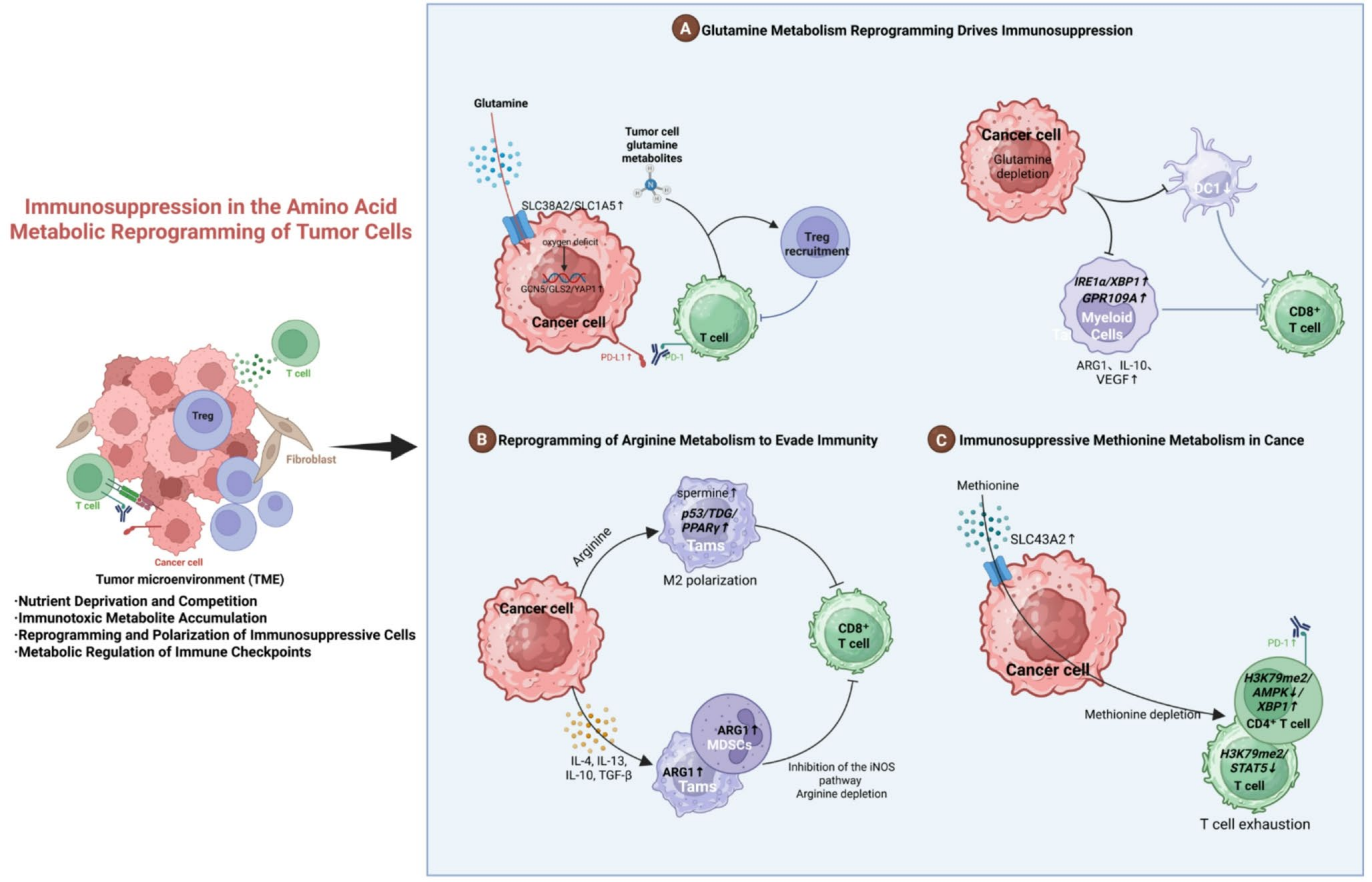
**A** Glutamine metabolism reprogramming drives immunosuppression: Tumor cells highly express transporters (e.g., SLC38A2, SLC1A5) that competitively deplete glutamine in the microenvironment. This not only impairs dendritic cell function and weakens antigen presentation but also induces endoplasmic reticulum stress in myeloid cells, polarizing them toward an immunosuppressive phenotype via the IRE1α/XBP1/GPR109A axis. Concurrently, active glutamine catabolism generates substantial ammonia byproducts, directly inducing effector T cell apoptosis and enriching regulatory T cells. Furthermore, under hypoxic conditions, the metabolic enzyme GLS2 can directly upregulate tumor cell PD-L1 expression through the GCN5/YAP1 axis, activating immune checkpoint inhibition, **B** Reprogramming of arginine metabolism to evade immunity: Tumor cell-secreted factors (IL-4, IL-13, IL-10, TGF-β, etc.) induce tumor-associated macrophages/myeloid-derived suppressor cells to overexpress arginase 1. This leads to arginine depletion in the microenvironment and inhibition of iNOS, directly resulting in T-cell dysfunction. Additionally, arginine secreted by cancer cells is taken up by macrophages and used to synthesize polyamines (spermine), which subsequently activates the p53/TDG signaling pathway. This drives the demethylation and transcriptional upregulation of the PPARγ gene, ultimately promoting the polarization of macrophages toward a pro-tumor M2-like phenotype, thereby strongly suppressing CD8⁺ T-cell function. **C** Immunoregulatory methionine metabolism in cancer: Tumor cells exhibit high expression of the transporter protein SLC43A2, which demonstrates uptake advantage in a low-methionine microenvironment, leading to T cells being subjected to “methionine starvation.” This reduces intracellular S-adenosylmethionine levels and specifically decreases histone H3K79me2 modification. The absence of this modification directly downregulates the transcription of the critical survival factor STAT5 in CD8⁺ T cells, triggering apoptosis and exhaustion; in CD4⁺ T cells, it induces endoplasmic reticulum stress by reducing AMPK expression, resulting in significant upregulation of PD-1 and driving the cells into a state of exhaustion. Treg: Regulatory T cells, TAMs: Tumor-Associated Macrophages, MDSCs: Myeloid-Derived Suppressor Cells, VEGF: Vascular Endothelial Growth Factor, ARG1: Arginase 1, IL-10: Interleukin-10, IL-4: Interleukin-4, IL-13: Interleukin-13, TGF-β: Transforming Growth Factor-beta. Created in https://BioRender.com

### Amino acid reprogramming and immune suppression: mechanisms of metabolic sabotage

#### Glutamine metabolism: competition, reprogramming, and immune checkpoint activation

Glutamine plays a central role in cancer cell metabolism, with its consumption rate second only to glucose, reflecting the tumor cell-specific phenomenon of “glutamine addiction”. This metabolic dependence stems from glutamine’s dual functions: on one hand, it serves as a nitrogen source for the synthesis of non-essential amino acids, purines, and pyrimidines; on the other hand, it enters the tricarboxylic acid cycle through its carbon skeleton, providing precursors and energy for biosynthesis (Jain et al. 2012). However, it must be explicitly noted that this phenomenon is only valid under conditions of standard culture media containing nonphysiologically high concentrations of cystine. Physiological levels of cystine in vivo can limit the replenishment of glutamine, whereas a high-cystine culture environment drives the uptake and breakdown of glutamine through the xCT/SLC7A11 transporter, thereby ‘manufacturing’ or exaggerating cellular glutamine dependence in vitro (Muir et al. 2017). Carcinogenic signals dominate this reprogramming process through precise regulation of transporter expression. c-Myc establishes a synergistic amino acid transport network by upregulating key transporters such as SLC1A5 (ASCT2), SLC7A5 (LAT1), systematically enhancing tumor cells’ uptake and utilization of glutamine (Gao et al. 2009). In contrast, in pancreatic cancer, KRAS mutations adopt a differential strategy by downregulating glutamate dehydrogenase (GLUD1) and upregulating transaminase GOT1, redirecting glutamine metabolism to a non-classical pathway to meet the specific demands of tumor cells under oxidative stress (Son et al. 2013).

This metabolic reprogramming not only supports tumor growth but also actively shapes an immunosuppressive microenvironment (Fig. 1). For instance, tumor cells compete with conventional dendritic cells (DCs) of type 1 in the tumor microenvironment for glutamine uptake by overexpressing the glutamine transporter SLC38A2, leading to “glutamine starvation” in the latter and impairing their ability to present antigens and activate CD8⁺ T cells (Guo et al. 2023). On the other hand, tumor cells produce a large amount of ammonia as a byproduct through active glutamine catabolism. After accumulation in the tumor microenvironment, ammonia mediates immunosuppression via two pathways: firstly, it directly induces apoptosis of CD8⁺ and CD4⁺ effector T cells; secondly, it enriches and enhances regulatory T cells (Tregs), further suppressing immune activity (Gu et al. 2026). In another study, hypoxia-induced glutamine metabolic reprogramming in pancreatic ductal adenocarcinoma can directly suppress T-cell function through the PD-1/PD-L1 pathway by activating the GCN5/GLS2 axis, using glutamate as a substrate to activate YAP1 transcriptional activity, and upregulating the expression of immune checkpoint protein PD-L1 (Chen et al. 2025). Furthermore, in hepatocellular carcinoma (HCC), cancer cells overexpress the glutamine transporter SLC1A5, competing with myeloid cells in the tumor microenvironment (TME) and extensively sequestering glutamine. The resulting glutamine deficiency disrupts the endoplasmic reticulum (ER) homeostasis of myeloid cells, activating the IRE1α/XBP1 signaling pathway in the unfolded protein response (UPR), thereby directly upregulating the expression of GPR109A. GPR109A further activates the downstream ERK signaling pathway, driving myeloid cells to express immunosuppressive factors such as Arg-1, VEGF, IL-10, and CXCL1, promoting their polarization toward an immunosuppressive phenotype and ultimately inhibiting the function of CD8 + T cells (Yang et al. 2025).

#### Arginine metabolism: ASS1 deficiency, immunosuppressive myeloid polarization, and T cell dysfunction

Arginine metabolic dysfunction is a significant metabolic abnormality in cancer, characterized by an “arginine nutritional deficiency” state resulting from the loss of function of Argininosuccinate synthase 1 (ASS1) (Gai et al. 2024). In non-small cell lung cancer (NSCLC), the activation of oncogenic KRAS mutations (e.g., G12D, G12C, G12V), which are common driver events in lung adenocarcinoma (Cancer Genome Atlas Research Network 2014), inhibits the expression of argininosuccinate synthase 1 (ASS1) through a histone deacetylase 3 (HDAC3)-dependent epigenetic reprogramming mechanism, thereby blocking the endogenous arginine synthesis pathway in cells. This ASS1 deficiency triggers profound metabolic remodeling, leading to aspartate accumulation (Gai et al. 2024). Studies have demonstrated that these aspartates are no longer utilized in the urea cycle but are redirected toward de novo synthesis of pyrimidine nucleotides to meet the precursors required for DNA replication, thereby promoting rapid proliferation of tumor cells (Gai et al. 2024). Concurrently, in colorectal cancer, arginine metabolic dysregulation occurs through another pathway: aberrant expression of arginase 1 (ARG1) is associated with reduced levels of β-hydroxybutyrylation, a post-translational modification that alters the interaction between ARG1 and the transporter SLC3A2, thereby disrupting intracellular arginine homeostasis and promoting tumor progression (Lin et al. 2025). The reprogramming of arginine metabolism by tumor cells not only meets their own proliferation needs but also actively shapes an immunosuppressive microenvironment (Fig. 1). The core mechanism lies in the fact that tumor-secreted factors (such as IL-4, IL-13, IL-10, and TGF-β) can induce tumor-associated macrophages (TAMs)and myeloid-derived suppressor cells (MDSCs) to overexpress arginase 1 (ARG1). Highly active ARG1, on one hand, competitively inhibits the tumor-inhibitory response mediated by inducible nitric oxide synthase (iNOS); on the other hand, it rapidly depletes arginine in the microenvironment. The depletion of arginine directly leads to downregulation of CD3ζ chain expression and cell cycle arrest in T cells, thereby fundamentally impairing their function (Rodriguez et al. 2017). Furthermore, in environments such as breast cancer, arginine secreted by cancer cells can be taken up by tumor-associated macrophages (TAMs)for active polyamine synthesis, leading to a significant increase in spermine levels. Elevated spermine acts as a key mediator to activate the p53 signaling pathway, thereby upregulating the expression and activity of thymidine DNA glycosylase. The latter catalyzes DNA demethylation at the nuclear receptor PPARγ gene locus, driving its transcriptional upregulation and ultimately promoting the polarization of macrophages toward a pro-tumor phenotype (M2 polarization). Polarized macrophages subsequently significantly inhibit the activation and effector functions of CD8 + T cells, mediating immune evasion (Zhu et al. 2025).

#### Tryptophan metabolism: The IDO/TDO-kynurenine-AhR axis in immune evasion

**Fig. 2:**
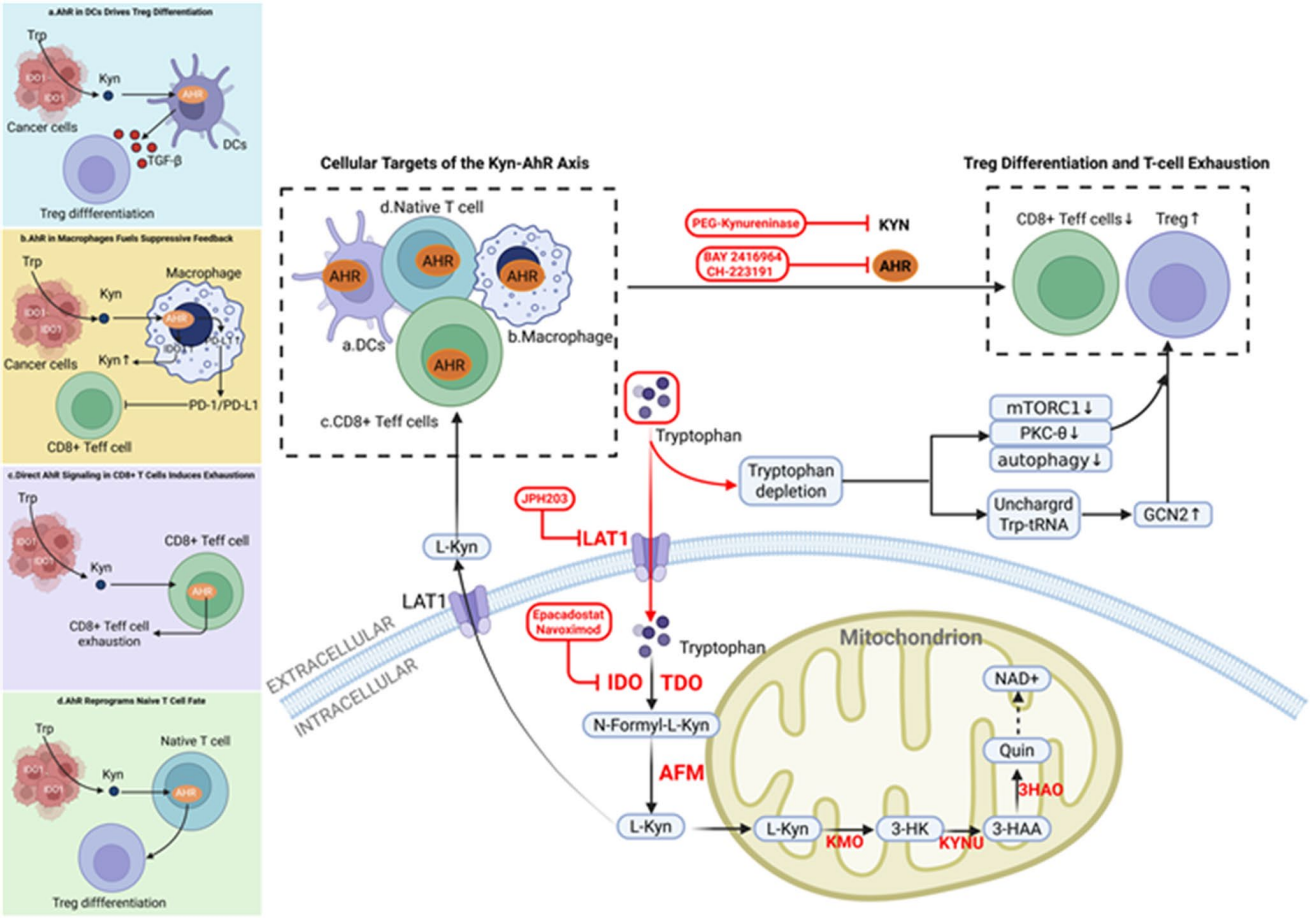
Tryptophan metabolism, Trp-Kyn-AHR axis mediating tumor immune escape and targeted therapeutic drugs. The expression of LAT1 receptors, IDO and TDO on the surface of tumor cells increases, enhancing their ability to metabolize tryptophan and generating a large amount of Kyn that is released outside the tumor cells. The free Kyn binds to dendritic cells, macrophages, CD8-positive T cells and AHR naive T cells, generating a strong immunosuppressive effect and promoting tumor immune escape. Furthermore, tryptophan depletion can directly activate the stress kinase GCN2 in CD8⁺ effector T cells, thereby inhibiting their function. *Trp* Tryptophan; *AHR* Aryl Hydrocarbon Receptor; *DC (s)*​ Dendritic Cell (s); *GCN2* General Control Nonderepressible 2 (a kinase); ​*IDO* Indoleamine 2,3-Dioxygenase; *Kyn* Kynurenine; *mTORC1* Mechanistic Target of Rapamycin Complex 1; *Teff* Effector T cell (typically refers to CD8 + Teff); *Treg* Regulatory T cell; *TDO* Tryptophan 2,3-Dioxygenase. Created in https://BioRender.com

The primary characteristic of tryptophan metabolic reprogramming in the tumor microenvironment is the abnormally high expression of key metabolic enzymes such as IDO1/IDO2 and TDO2 (Munn and Mellor 2007). The overactivation of these enzymes leads to significant depletion of tryptophan in the microenvironment, accompanied by substantial accumulation of immunosuppressive metabolites such as kynurenine (Kyn), thereby forming an immunosuppressive microenvironment (Opitz et al. 2011). For instance, the tryptophan (Trp) metabolic pathway in the tumor microenvironment (TME) is a key mechanism mediating immune evasion. In this process, indoleamine 2,3-dioxygenase 1 (IDO1) and tryptophan 2,3-dioxygenase (TDO) catalyze Trp to kynurenine (Kyn), thereby triggering a dual immunosuppressive effect: on one hand, Trp depletion activates the stress kinase GCN2 in T cells, inhibiting effector T cell function and inducing apoptosis; on the other hand, Kyn accumulation activates the aryl hydrocarbon receptor (AhR), promoting the differentiation of regulatory T cells (Tregs) and myeloid suppressor cells, and enhancing IDO1 expression through the IL-6/STAT3 positive feedback loop, forming a vicious cycle (Fujiwara et al. 2022). To date, a variety of drugs targeting tryptophan metabolism reprogramming and the tumor immune evasion it mediates have been developed. Inhibitors such as the IDO inhibitors epacadostat and navoximod, the LAT1 inhibitor JPH203, and the AHR inhibitors BAY 2,416,964 and CH-223,191 will be discussed in detail below (Fig. 2).

#### Methionine metabolism: epigenetic silencing of T cells and activation of innate immunity

The “Hoffmann effect” exhibited by tumor cells, characterized by their specific dependence on exogenous methionine, stems from inherent defects in the metabolic network of tumor cells. Upstream oncogenes (e.g., MYC) promote the conversion of methionine to S-adenosylmethionine (SAM) by regulating the expression of the transporter SLC43A2 and the key enzyme MAT2A (Bin et al. 2024).

Notably, this methionine metabolic reprogramming not only benefits the tumor itself but also actively shapes an immunosuppressive microenvironment (Fig. 1). In various tumor cells (such as melanoma B16F10 and colon cancer MC38/CT26), the high expression of the methionine transporter SLC43A2 confers an uptake advantage to tumor cells in a low-methionine microenvironment, leading to “methionine starvation” of T cells. This starvation state reduces the level of S-adenosylmethionine (SAM) within T cells, thereby specifically decreasing the histone H3K79me2 modification. The depletion of H3K79me2 directly inhibits the transcription of the key signaling molecule STAT5, triggering T cell apoptosis and functional failure, ultimately weakening anti-tumor immunity (Bian et al. 2020). Furthermore, in CD4⁺ T cells, the downregulation of H3K79me2 similarly weakens its binding to the promoter of the Prkaa1 gene (encoding AMPKα1), leading to reduced AMPK expression. The decrease in AMPK triggers endoplasmic reticulum stress, activating XBP1, a key factor in the unfolded protein response, ultimately resulting in a significant elevation of PD-1 protein levels on the cell surface, driving CD4⁺ T cells into exhaustion (Pandit et al. 2023).

### Author’s perspective: key insights and future directions

Although numerous previous reviews have extensively elaborated on the amino acid metabolic dependence of tumor cells and their potential as therapeutic targets, most discussions exhibit two critical limitations: first, they typically focus on the metabolic vulnerability of tumor cells themselves, while systematic elaboration on how metabolic reprogramming can serve as an active “weapon” to dismantle anti-tumor immunity remains insufficient; second, there is a lack of integrated analysis of the profound challenges faced by strategies targeting these metabolic axes (e.g., drug resistance due to metabolic plasticity, complex impacts on the immune microenvironment, etc.). This review aims to fill these gaps and provides the following key advancements and new syntheses: (1) Unified Framework: We systematically evaluate the four major metabolic axes—glutamine, arginine, tryptophan, and methionine—within a unified “metabolism-immune dialogue” framework; (2) Critical Evaluation: This article not only lists therapeutic outcomes but also critically assesses the double-edged sword effect of various targeting strategies (e.g., enzyme inhibitors, prodrug compounds, dietary restrictions) between “tumor killing” and “immune reprogramming,” with dedicated discussions on their limitations (e.g., resistance mechanisms, immunogenicity, off-target toxicity) to reveal core barriers from preclinical translation to clinical success; (3) Prospective on Combination Strategies: We particularly emphasize and analyze the immense potential and biological basis of combination therapy strategies based on metabolism (e.g., synergies between metabolic inhibitors and immune checkpoint blockers).

## Therapeutic targeting of amino acid metabolic axes: mechanisms and agents

Against this backdrop, this review systematically elucidates the mechanisms and research progress of the emerging anti-tumor strategy targeting the amino acid metabolic axis. Targeting the amino acid metabolic axis not only exhibits significant antitumor effects but also reshapes tumor cell-mediated immune suppression through amino acid metabolic reprogramming, offering broad prospects for this field. The article focuses on investigating targeted inhibition strategies for four key amino acids (glutamine, arginine, tryptophan, and methionine) in the metabolic axis.

### Targeting glutamine metabolism: deprivation and inhibition strategies

#### Research on glutamine depletion and transport inhibition

Previous studies have demonstrated that V-9302 exhibits therapeutic potential in various cancer models by inhibiting the substrate transport of ASCT2 (including glutamine) through the blockade of the ASCT2 transporter. Its mechanism of action involves blocking glutamine uptake, thereby depleting intracellular glutathione and inducing reactive oxygen species (ROS) accumulation, ultimately triggering glutamine-dependent apoptosis in tumor cells. In xenograft models, V-9302 monotherapy effectively inhibits tumor growth (Schulte et al. 2018). However, it should be explicitly noted that V-9302 is not a highly selective ASCT2 inhibitor. Subsequent studies have demonstrated its efficacy in inhibiting multiple amino acid transporters, including SNAT2 (SLC38A2). In fact, pharmacological studies targeting ASCT2 have long faced the challenge of poor inhibitor selectivity (Bröer 2018). In head and neck squamous cell carcinoma (HNSCC), overexpression of ASCT2 is associated with poor prognosis. The combination of ASCT2 knockdown (e.g., by shRNA) and pharmacological SNAT2 inhibition (using the small-molecule antagonist V-9302)​ reduces intracellular glutamine levels and disrupts glutathione synthesis, thereby inhibiting cell proliferation and enhancing oxidative stress and mTORC1 pathway suppression (Zhang et al. 2020). Additionally, the combination of V-9302 with the MEK inhibitor trametinib in non-small cell lung cancer (NSCLC) induces GSDME-dependent pyroptosis and cell cycle arrest by regulating the FOXO3a/FOXM1 axis and autophagy, significantly enhancing in vivo antitumor efficacy (Liu et al. 2025). In terms of immunomodulation, V-9302 selectively inhibits glutamine uptake in triple-negative breast cancer (TNBC) tumor cells without affecting CD8 + T cell function, thereby improving anti-tumor T cell activity by maintaining glutathione balance (Edwards et al. 2021). The above evidence collectively indicates that selective targeting of glutamine transport via V-9302 represents a promising anti-tumor strategy with broad prospects.

Targeting glutaminase (GLS), a key enzyme in the glutamine metabolic pathway, has demonstrated significant therapeutic potential (Altman et al. 2016). Lukey et al. (2016) demonstrated that in breast cancer, oncogenic signals (e.g., Rho GTPase) upregulate glutaminase (GLS) expression via the JNK/c-Jun pathway, conferring tumor cell dependence on glutamine metabolism. This study further showed that targeting this metabolic vulnerability with the GLS inhibitor CB-839 effectively suppresses tumor growth. In hepatocellular carcinoma (HCC), GLS1 expression is significantly upregulated and closely associated with tumor progression. CB-839 exerts dose-dependent cytotoxic effects by inhibiting glutamine catabolism, reducing ammonia production, and increasing the glutamine/glutamate ratio (a 46% survival rate reduction at 10 µM). This mechanism involves the deprivation of α-ketoglutarate, a key substrate of the TCA cycle, with minimal impact on normal hepatocytes, exhibiting favorable selective toxicity (Tambay et al. 2025). In drug-resistant liver cancer models, CB-839 also enhances 5-FU sensitivity by inducing ROS accumulation and glutathione depletion, thereby triggering oxidative stress (Wang et al. 2026).

In combination therapy, CB-839 demonstrates significant synergistic effects with multiple targeted agents. In high-grade serous ovarian cancer (HGSOC), CB-839 in combination with the thioredoxin reductase inhibitor auranofin exhibits synergistic antitumor activity against MYC-high tumors (Raninga et al. 2023). In multiple myeloma (MM), CB-839 not only inhibits cell proliferation alone but also significantly enhances sensitivity to histone deacetylase (HDAC) inhibitors, triggering apoptosis through cross-talk between metabolic reprogramming and epigenetic regulation (Okabe et al. 2024).

**Fig. 3:**
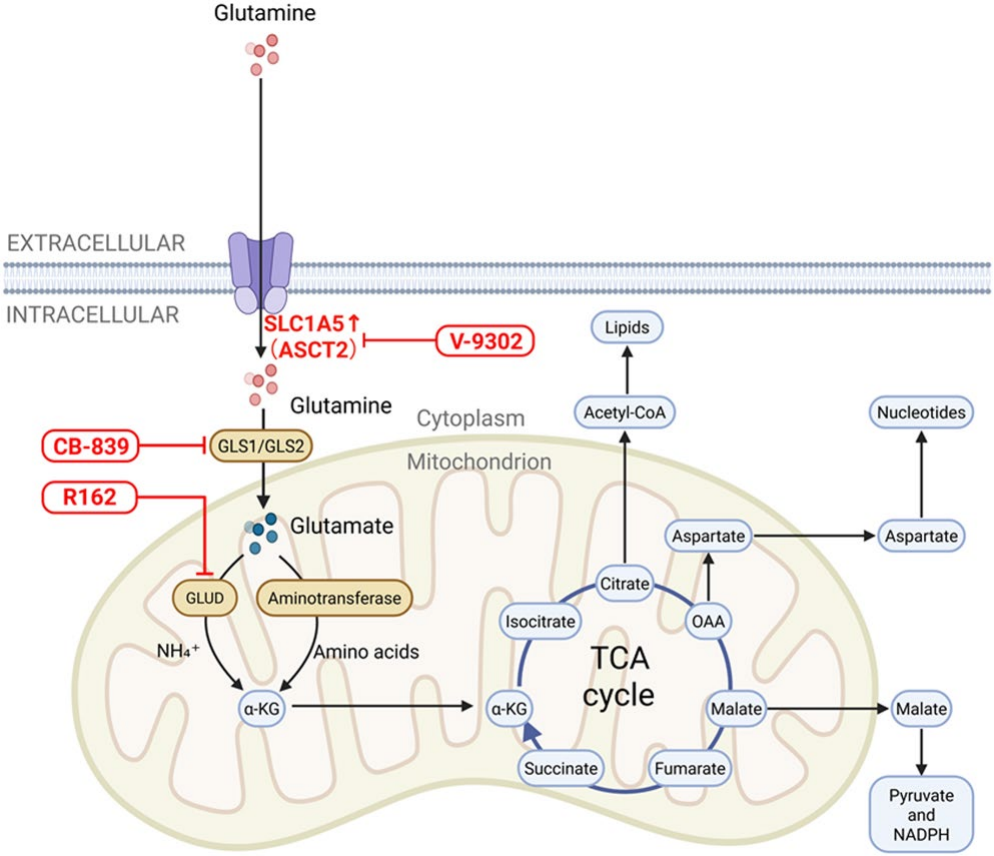
Glutamine enters tumor cells via the transporter protein ASCT2 and is subsequently degraded into glutamic acid by glutaminase. Glutamic acid is then converted into α-ketoglutarate through the catalysis of glutamate dehydrogenase or transaminase, thereby entering the tricarboxylic acid cycle to provide energy and carbon skeletons for tumor cell proliferation. During this process, intermediate products in the cycle are utilized for critical biosyntheses: (1) Citrate is transported from mitochondria to the cytoplasm, where it is cleaved into oxaloacetate and acetyl-CoA, which participate in lipid metabolism; (2) Oxaloacetate undergoes transamination to generate aspartate, which is involved in nucleotide synthesis; (3) Malate is converted into pyruvate, simultaneously producing NADPH. *Acetyl-CoA* Acetyl-Coenzyme A, *α-KG* Alpha-Ketoglutarate, *OAA* Oxaloacetate, *NADPH* Nicotinamide Adenine Dinucleotide Phosphate, *GLS1/GLS2* Glutaminase 1 / Glutaminase 2, *GLUD* Glutamate Dehydrogenase, *TCA cycle* Tricarboxylic Acid Cycle. Created in https://BioRender.com

Mechanistic studies have demonstrated that CB-839 monotherapy triggers compensatory resistance mechanisms. In pancreatic cancer, CB-839 activates the GCN2-ATF4 signaling pathway, and its combination with the ASCT2 inhibitor V-9302 overcomes this adaptive resistance, resulting in synergistic anticancer effects (Kim et al. 2025). In head and neck squamous cell carcinoma (HNSCC), CPI-613 is a lipoate analog that inhibits mitochondrial enzymes pyruvate dehydrogenase and α-ketoglutarate dehydrogenase, with monotherapy capable of compensatorily upregulating GLS1. The combination of CPI-613 with the glutaminase inhibitor CB-839 generates a “double metabolic blow,” synergistically blocking this metabolic redundancy and exerting a stronger anticancer effect (Lang et al. 2021). In non-small cell lung cancer (NSCLC), the combination of the glutaminase (GLS1) inhibitor CB-839​ and the cyclin-dependent kinase 7 (CDK7) inhibitor THZ1​ simultaneously targets two key metabolic pathways. THZ1 monotherapy blocks the glycolysis pathway in cancer cells, while CB-839 inhibits glutamine metabolism. Their concurrent application disrupts both glucose and glutamine utilization, thereby generating synergistic antitumor activity (Cheng et al. 2019).

Furthermore, in colorectal cancer (CRC), CB-839 exerts multiple inhibitory effects on the tumor microenvironment by suppressing cancer cell proliferation, stromal activation, tumor migration, and angiogenesis (Miyamoto et al. 2024). These studies collectively demonstrate that CB-839, as a key drug targeting glutamine metabolism, exhibits significant therapeutic value in both monotherapy and combination strategies.

The GLUD1 inhibitor R162 exerts its therapeutic effects by targeting the reprogramming of glutamine metabolism in tumor cells. This inhibitor reverses tumor metastasis and drug resistance mediated by epithelial-mesenchymal transition (EMT) (Wang et al. 2022). In a model of acquired EGFR TKI-resistant non-small cell lung cancer (NSCLC), R162 effectively inhibited tumor progression (Qu et al. 2025). Studies have demonstrated that this inhibitor also exhibits significant efficacy in LKB1-deficient lung cancer (Jin et al. 2018). Therefore, based on existing research (Wang et al. 2022; Qu et al. 2025; Jin et al. 2018), targeting GLUD1-mediated glutamine metabolic reprogramming may represent a highly promising novel strategy for future cancer treatment (The relevant mechanism is shown in Fig. 3).

Traditional DON has been hindered in clinical translation due to its severe gastrointestinal toxicity. To address this, researchers have developed tumor-targeted prodrug strategies, where such prodrugs remain inert in systemic circulation but are specifically cleaved by enzymes in the tumor microenvironment to release active DON, thereby significantly reducing toxicity. Among these, JHU083 and DRP-104 are representative drugs. JHU083 comprehensively inhibits tumor cells ‘glycolysis, tricarboxylic acid cycle, pentose phosphate pathway, and purine synthesis, triggering energy and redox crises. It also induces metabolic collapse and growth inhibition by downregulating c-MYC and activating AMPK. Simultaneously, JHU083 upregulates T cells’ oxidative phosphorylation and reserve respiratory capacity, enhancing their ability to utilize acetate and replenish metabolism through pyruvate carboxylation, thereby gaining functional advantages under metabolic stress (Leone et al. 2019). Another representative drug, DRP-104, is also designed based on the prodrug principle. As a broad-spectrum inhibitor of glutamine, it not only suppresses tumors by reducing the levels of intermediate products in the tumor tricarboxylic acid cycle and purine synthesis but also significantly reverses immunosuppression. Specifically, DRP-104 promotes the infiltration of CD8⁺ T cells into tumors and induces their phenotypic transformation into stem cell-like central memory cells, while simultaneously reducing the expression of exhaustion markers. Additionally, DRP-104 exhibits potent synergistic effects with anti-PD-1 immunotherapy, significantly improving patient survival rates (Rais et al. 2022).

#### Challenges and limitations in targeting glutamine metabolism

Although drugs targeting glutamine metabolism have demonstrated significant efficacy in antitumor and immunosuppression reversal, inhibiting a single node often fails to completely block tumor progression. For instance, in a c-MYC-driven liver cancer model, specific knockout of glutaminase 1 (GLS1) only delayed rather than prevented tumorigenesis, an effect that could be offset by upregulation of its isoenzyme GLS2. Even when both GLS1 and GLS2 are simultaneously knocked down, tumor cells can still maintain glutamate production by activating transaminases and utilizing glutamine as an amide nitrogen donor. Concurrently, tumor cells rapidly enhance glycolysis to compensate for energy loss and precursor synthesis depletion through glucose-derived carbon sources (Méndez-Lucas et al. 2020). Furthermore, the study revealed that long-term CB-839 treatment in pancreatic ductal adenocarcinoma induces tumor cells to utilize branched-chain fatty acids as an alternative energy source and carbon source. Concurrently, cells upregulate multiple antioxidant proteins (e.g., CTH, ALH1L2) to counteract the reduction of reductants (NADPH, GSH) and accumulation of reactive oxygen species (ROS) caused by impaired glutamine metabolism. Additionally, the expression of glutamine-dependent enzyme asparaginase synthetase (ASNS) and glutamine-independent enzymes branched-chain amino acid transaminase 1 (BCAT1) and alanine transaminase 2 (GPT2) is significantly elevated, thereby restoring glutamate supply through multiple pathways. Based on these compensatory mechanisms, targeting the corresponding pathways demonstrates promising therapeutic potential: studies have confirmed that the combined use of bis (2-sulfopropionyl)oxybenzene (BSO) to inhibit γ-glutamylcysteine synthetase (GCLC/GCLM) and CB-839 effectively blocks cell regeneration after CB-839 treatment and significantly inhibits tumor progression in in vivo models (Biancur et al. 2017). These adaptive responses collectively reveal the remarkable metabolic plasticity of tumor cells. On the other hand, prodrug strategies aimed at reducing toxicity have significantly improved safety, but they cannot completely eliminate off-target effects. For example, although the prodrug DRP-104 of the broad-spectrum glutamine antagonist DON exhibits markedly improved toxicological characteristics compared to DON, mild lymphocytic infiltration was observed in gastrointestinal tissues at the highest dose required for optimal antitumor efficacy (1 mg/kg). This indicates that even at optimized doses achieving highly tumor-targeted delivery, the therapy still induces mild local immune-inflammatory responses, providing direct pathological evidence for residual off-target toxicity (Rais et al. 2022).

### Arginine deprivation therapy: exploiting ASS1 deficiency

#### Clinical and preclinical agents for arginine depletion

ADI-PEG20 is a polyethylene glycol-modified arginine deaminase that catalyzes the conversion of plasma arginine to citrulline and ammonia, thereby depleting arginine levels in the blood. This drug selectively targets ASS1-deficient tumor cells, which cannot synthesize arginine due to the absence of Argininosuccinate synthase, resulting in an “arginine starvation” effect (Beddowes et al. 2017). In preclinical studies of ASS1-low expression pancreatic ductal adenocarcinoma, ADI-PEG20 demonstrated significant synergistic effects when combined with the histone deacetylase (HDAC) inhibitor palbociclib. In xenograft models, the combination therapy led to a substantial reduction in tumor volume (75% reduction in the MiaPaca2 model and 67% reduction in the Patu8988t model), outperforming monotherapy (Kim et al. 2020). In a phase I dose-escalation trial of ASS1-deficient breast cancer, the ADI-PEG20 regimen combined with cisplatin and pemetrexed showed good safety without dose-limiting toxicity. This regimen continuously depletes plasma arginine and achieved objective response in refractory sarcomatoid pleural mesothelioma (Beddowes et al. 2017). Another Phase I study demonstrated that ADI-PEG20 combined with cisplatin was well-tolerated in patients with advanced solid tumors, achieving a disease control rate of 46% (Yao et al. 2021). In a Phase II study of relapsed/refractory acute myeloid leukemia, the complete response rate with ADI-PEG20 monotherapy was 4.7%, while the disease control rate reached 42.9% in evaluable patients. Two patients who achieved remission had response durations of 7.5 and 8.8 months, suggesting potential benefits in some patients (Tsai et al. 2017). The ATOMIC-Meso Phase III trial confirmed that in patients with non-epithelial pleural mesothelioma, ADI-PEG20 combined with chemotherapy significantly improved overall survival (9.3 months vs. 7.7 months) compared to chemotherapy alone, with a 29% reduction in mortality risk. The 36-month survival rate in the combination therapy group was nearly 12%, nearly three times that of the control group (Szlosarek et al. 2024). As a novel therapy targeting tumor metabolism, ADI-PEG20 has shown definitive efficacy in tumors with ASS1 deficiency. Evidence from preclinical to Phase III studies indicates that it exhibits synergistic antitumor effects when combined with standard chemotherapy, providing a new therapeutic option for refractory tumors.

BCT-100 (polyethylene glycolylated recombinant human arginase) selectively targets arginine-deficient tumor cells that cannot synthesize arginine due to defects in the ASS1 or OTC enzymes by catalyzing the degradation of plasma arginine, inducing cell cycle arrest (e.g., G1 phase) and apoptosis (Fung et al. 2025). In preclinical studies of malignant pleural mesothelioma (MPM), the drug demonstrated dose-dependent proliferation inhibition in multiple cell lines and significant tumor growth suppression in animal models (Lam et al. 2017). Similarly, in acute myeloid leukemia (AML), BCT-100 exerts cytotoxic effects on cell lines and primary cells by depleting arginine, synergizing with chemotherapeutic agents such as cytarabine to enhance anti-leukemic effects (Mussai et al. 2015). Additionally, in one case, it overcame immune checkpoint inhibitor resistance in melanoma, inducing durable response (De Santo et al. 2018), and showed synergistic effects with standard chemotherapy in neuroblastoma (Hanssen et al. 2025). Early clinical data indicate favorable tolerability of BCT-100, providing strong support for its further development as a monotherapy or combination therapy for various solid tumors and hematologic malignancies.

#### Limitations and adaptive resistance in arginine-targeted therapy

Therapeutic strategies targeting arginine metabolism still face significant limitations. For instance, the efficacy of ADI-PEG20 is strictly dependent on the ASS1 deficiency in tumor cells, rendering it ineffective against tumors with high ASS1 expression, which fundamentally restricts its applicable patient population. Secondly, tumor cells exhibit robust metabolic adaptability: even in the absence of ASS1 deficiency, arginine starvation induced by ADI-PEG20 triggers upregulation and enhanced membrane localization of the CAT-1 transporter, thereby accelerating the uptake of exogenous arginine and forming a critical adaptive resistance (Prudner et al. 2018). Furthermore, in a phase II randomized clinical trial involving patients with ASS1-deficient malignant pleural mesothelioma, ADI-PEG20 as an exogenous protein drug elicited neutralizing antibodies in patients. Notably, the duration of arginine deprivation was significantly positively correlated with progression-free survival, confirming that immunogenicity is one of the key mechanisms compromising its therapeutic sustainability (Szlosarek et al. 2024).

### Intervention in tryptophan metabolism: disrupting the IDO/TDO-Kyn-AhR axis

#### Pharmacological inhibitors of the tryptophan-kynurenine pathway

JPH203, as a highly selective LAT1 (SLC7A5) inhibitor, has demonstrated broad antitumor potential in various cancer models. In prostate cancer studies, JPH203 effectively reverses cabazitaxel resistance by inhibiting the LAT1-CDK1/2 axis, significantly reducing cell migration and invasion capabilities, and downregulating the expression of epithelial-mesenchymal transition (EMT) markers, thereby suppressing tumor progression (Rii et al. 2024). In acute myeloid leukemia (AML), JPH203 alone induces apoptosis and reduces mitochondrial oxidative phosphorylation (OXPHOS) capacity, while exhibiting synergistic effects when combined with venetoclax.

and azacitidine (Ven + Aza), disrupting branched-chain amino acid metabolism and energy homeostasis (Zhang et al. 2023). For triple-negative breast cancer (TNBC), JPH203 inhibits cell proliferation dose-dependently, induces G2/M phase arrest and apoptosis, and reshapes the immune microenvironment (e.g., increasing CD8 + T cell infiltration), producing synergistic antitumor effects when combined with PD-1 antibodies (Zhao et al. 2025). In thyroid cancer, JPH203 significantly inhibits tumor growth in both in vitro and in vivo models by blocking the mTORC1 signaling pathway, with low cytotoxicity to normal cells (Hafliger et al. 2018; Enomoto et al. 2019). These studies collectively demonstrate that JPH203 exerts therapeutic effects in various malignancies by targeting LAT1-mediated amino acid metabolic reprogramming, particularly showing synergistic potential when combined with standard chemotherapy or immunotherapy.

Preclinical studies have demonstrated the promising therapeutic potential of IDO inhibitors. The study by Watanabe et al. (2020) showed that indoximod combined with high-dose fractionated radiotherapy (hRT) and immune checkpoint inhibitors could induce rapid tumor regression in animal models through synergistic activation of NK cells and CD8 + T cells, with this synergistic effect remaining consistent when different checkpoint inhibitors (anti-PD-1/anti-CTLA-4) were used. Furthermore, in specific populations such as pediatric brain tumors, indoximod combined with chemotherapy has demonstrated unique clinical value (Johnson et al. 2024), extending patient survival (median OS 13.3 months) with favorable safety profiles. These findings suggest that tumor microenvironment heterogeneity and treatment timing may be critical factors influencing therapeutic efficacy.

As previously described, tryptophan metabolic reprogramming mediates tumor immune evasion. Multiple strategies have been developed targeting this pathway. For example, the combination of IDO inhibitors (such as indoximod) with the anti-PD-1 antibody pembrolizumab achieved an objective response rate (ORR) of 51% in a phase II trial of advanced melanoma, with good safety (Zakharia et al. 2021). In cervical cancer models, LAMP3 + dendritic cells, which highly express IDO1, form a PD-1/PD-L1-mediated immunosuppressive network with T cells. While the IDO inhibitor epacadostat alone was ineffective, its combination with anti-CTLA-4 significantly inhibited tumor growth and restored CD8 + T cell proliferation (Ki67+) and cytotoxicity (granulase B+) (Qu et al. 2023). Furthermore, the enzyme therapy PEG-Kynureninase can directly degrade Kyn. In models such as melanoma and colon cancer, it alone enhances CD8 + T cell infiltration and multipotency. When combined with anti-PD-1 or anti-CTLA-4 agents, it can increase the complete response rate to 45–60% (Triplett et al. 2018). Downstream AhR inhibitors (e.g., CH-223191) reduce the M1 phenotype of Tregs and reprogrammed macrophages by blocking the Kyn-AhR axis, and synergistically prolong survival with anti-PD-1 agents (Campesato et al. 2020). These studies highlight the broad prospects of targeting the Trp-Kyn-Ah pathway in combination with immune checkpoint inhibitors for reversing immunosuppression and enhancing anti-tumor immunity.

#### Clinical translation challenges of tryptophan metabolism inhibitors

Targeting the tryptophan metabolic pathway in therapy still faces some key clinical translation challenges. In the study by Long et al. (2019), the combination regimen of the IDO inhibitor epacadostat with pembrolizumab did not improve progression-free survival (HR = 1.00) in patients with melanoma. This negative outcome from the pivotal Phase III clinical trial highlights the common clinical translation challenges faced by IDO inhibitors (such as epacadostat and navoximod) when combined with immune checkpoint inhibitors, with most failing to demonstrate significant advantages. Similarly, the study of navoximod combined with atezolizumab (Jung et al. 2019) and the study of epacadostat combined with pembrolizumab in sarcoma (Kelly et al. 2023) both achieved only limited response rates (ORR 3.3–11%), highlighting the clinical translation challenges of IDO inhibitors in advanced solid tumors. The contradictory results highlight the complexity of translational medicine, underscoring the need for future efforts in biomarker development, optimization of delivery strategies, and elucidation of compensatory mechanisms to achieve precise individualized immunotherapy. Furthermore, a study revealed that the amino acid transporter LAT1 on the blood-brain barrier (BBB) is critical for maintaining normal levels of branched-chain amino acids (BCAAs) in the brain, particularly leucine and isoleucine. Defects in its function (e.g., genetic mutations) can lead to BCAA deficiency in the brain, which subsequently activates the amino acid response (AAR) pathway, inhibits protein translation, disrupts inhibitory synaptic transmission, and ultimately results in autism-like behaviors and motor impairments (Tărlungeanu et al. 2016). Therefore, based on this mechanism, drugs such as JPH203, which target the inhibition of LAT1 function, may carry similar risks of neurological and motor dysfunction.

### Manipulating methionine metabolism: from dietary restriction to synthetic lethality

#### Strategies for methionine restriction and enzyme inhibition

Methionine, as a sulfur-containing essential amino acid, plays multiple critical roles in cellular metabolism: it serves not only as a precursor for the biosynthesis of succinyl-CoA, glutathione, and creatine, but also drives epigenetic modifications such as DNA methylation and regulates gene expression by acting as a core methyl donor through its active form, S-adenosylmethionine (SAM) (Martinez et al. 2017). SAM plays a pivotal role in maintaining the epigenetic state of cells (Ducker and Rabinowitz 2017). Its rapid depletion renders tumor cells highly dependent on exogenous methionine to sustain their malignant phenotype (Wang et al. 2019).

The unique “methionine dependence” of cancer cells—i.e., their inability to utilize homocysteine as a substitute for methionine—stems from their methyl metabolic imbalance (“methyl sink”), which provides a breakthrough for targeted therapy (Martinez et al. 2017). Studies have shown that dietary methionine restriction (MR) can reduce cGAS methylation by modulating the “methionine-SUV39H1-UHRF1 axis,” activate the cGAS-STING innate immune pathway, promote type I interferon production, and reshape the anti-tumor immune microenvironment (Wei and Locasale 2023). Specifically, the reduction in SAM levels caused by methionine restriction can attenuate the methylation modification of cGAS by SUV39H1. The demethylation of cGAS activates its STING signaling pathway, promoting the production of type I interferon in tumor cells, thereby reversing the “cold” tumor microenvironment into a “hot” microenvironment and significantly increasing the infiltration of various immune cells, including CD8⁺ T cells. Preclinical studies have demonstrated that methionine restriction or SUV39H1 inhibitors (Chaetocin) alone can inhibit tumor growth and enhance immune infiltration. Furthermore, both agents exhibit synergistic antitumor effects when combined with radiotherapy or anti-PD-1 therapy (Fang et al. 2023). Additionally, another study found that SAM depletion induced by methionine restriction reduces the overall RNA m6A modification level in tumor cells, thereby decreasing the recognition and binding of the m6A “reader” protein YTHDF1 to specific mRNAs. The reduced binding of YTHDF1 directly inhibits the translation efficiency of these mRNAs, leading to downregulation of PD-L1 and VISTA protein expression. As a result, the inhibitory effect of tumor cells on CD8⁺ T cells is relieved, thereby restoring the antitumor function of T cells. In animal models, methionine restriction combined with anti-PD-1 antibodies demonstrated significant synergistic efficacy (Li et al. 2023).

In therapeutic applications, MR demonstrates broad synergistic potential: in colorectal cancer models, MR combined with low-dose 5-FU exhibits potent synergy in inhibiting tumor growth; in soft tissue sarcomas, MR combined with radiotherapy extends the tumor doubling time by 52% (Gao et al. 2019); in pancreatic cancer, MR upregulates TRAIL-R2 expression, enhancing the sensitivity of TRAIL agonists to induce apoptosis (Yamamoto et al. 2020). Furthermore, MR monotherapy demonstrated survival benefits comparable to chemotherapy with lower toxicity in metastatic colon cancer, ovarian cancer, and renal cell carcinoma models (Jimenez-Alonso et al. 2023). These findings highlight MR as a metabolic intervention strategy that can form a “synthetic lethality” effect with chemotherapy, radiotherapy, and targeted therapies through multiple mechanisms such as immune activation, epigenetic regulation, and apoptosis sensitization, providing a novel adjuvant treatment paradigm with clear translational prospects for advanced cancers (particularly drug-resistant ones).

**Fig. 4:**
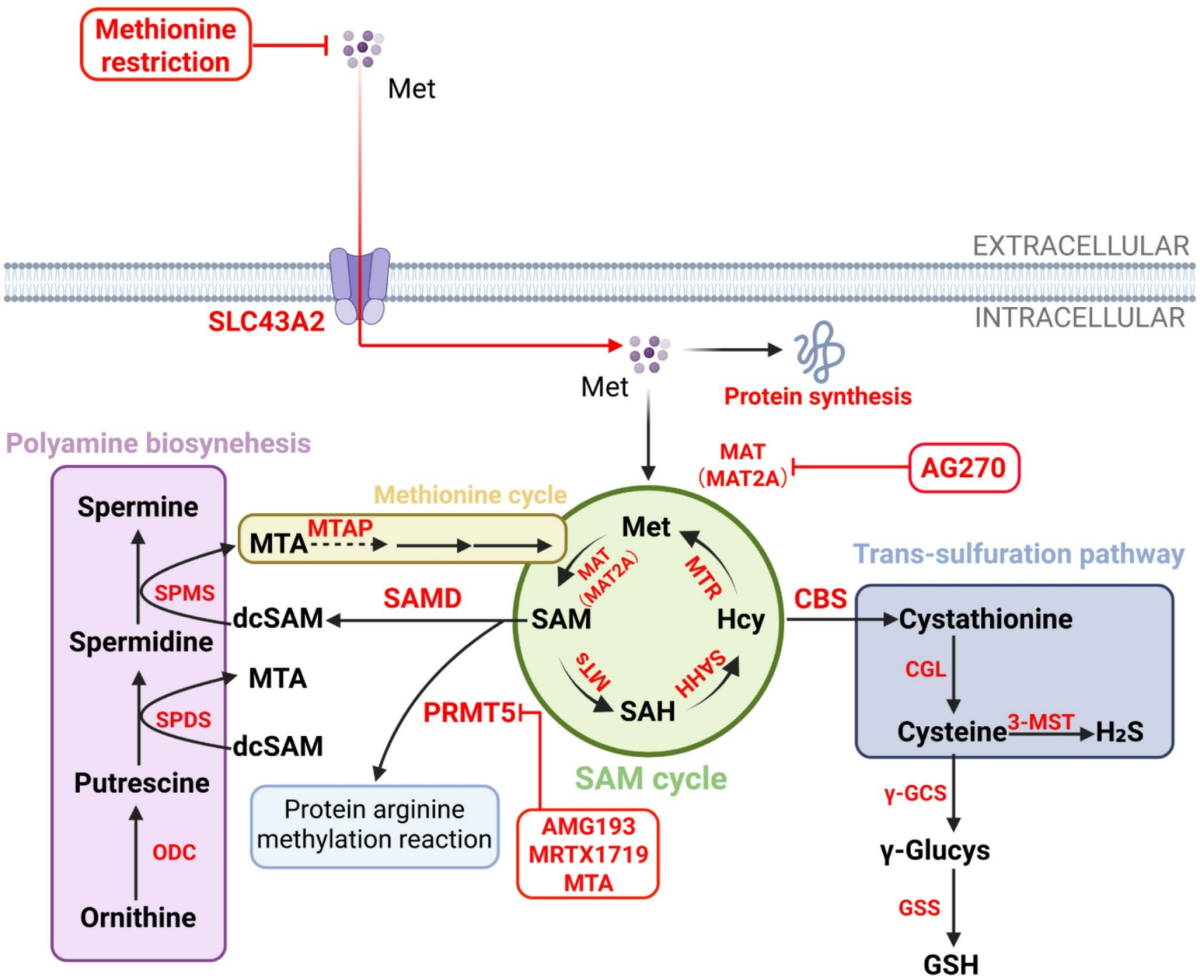
In tumor cells, methionine is transported into the cell via the transporter SLC43A2, subsequently participating in two critical biological processes: direct involvement in protein synthesis and entry into the S-adenosylmethionine (SAM) cycle. In this cycle, methionine is first catalyzed by adenosyltransferase to form S-adenosylmethionine (SAM). SAM serves as the most important methyl donor in cells, where it can transfer methyl groups to target proteins via methyltransferases such as PRMT5, participating in protein arginine methylation. This process plays a pivotal role in epigenetic regulation and signal transduction in tumor cells. Additionally, SAM can serve as a substrate for polyamine synthesis. In this pathway, SAM is metabolized to 5’-methylthioadenosine (MTA), which competitively inhibits PRMT5, thereby exposing the sensitivity and vulnerability of tumor cells to PRMT5 inhibitors. *Met* Methionine; *ODC* Ornithine Decarboxylase; *SPDS* Spermidine Synthase; *SPMS* Spermine Synthase; *MTAP* Methylthioadenosine Phosphorylase; *MTA* 5’-Methylthioadenosine; *SAM* S-Adenosylmethionine; *SAH* S-Adenosylhomocysteine; *​MAT* Methionine Adenosyltransferase; *MTR* Methionine Synthase; *CBS* Cystathionine Beta-Synthase; *CGL* Cystathionine Gamma-Lyase; *3-MST* 3-Mercaptopyruvate Sulfurtransferase; *H₂S* Hydrogen Sulfide; *γ-GCS* γ-Glutamylcysteine Synthetase; *γ-GluCys* γ-Glutamylcysteine; *GSS* Glutathione Synthetase; *GSH* Glutathione. Adapted from Bin et al. *Trends Pharmacol. Sci.*, 2024. Created in https://BioRender.com

Among various malignant tumors, homozygous deletion of the methionine adenosine phosphatase (MTAP) gene constitutes a critical metabolic vulnerability (Kalev et al. 2021). This defect leads to the accumulation of its substrate 5’-methylthioadenosine (MTA), which partially inhibits the activity of S-adenosylmethionine (SAM)-dependent enzyme protein arginine methyltransferase 5 (PRMT5), rendering cancer cells abnormally sensitive to further reductions in intracellular SAM levels (Konteatis et al. 2021). Targeting this weakness, methionine adenosine transferase 2 A (MAT2A), as a key rate-limiting enzyme catalyzing SAM synthesis (Li et al. 2022), has emerged as an ideal pharmacological intervention target. Studies have demonstrated that oral MAT2A inhibitors (e.g., AG-270) can selectively and potently inhibit PRMT5 activity in MTAP-deficient cancer cells by precisely reducing intracellular SAM levels in synergy with endogenous MTA (Kalev et al. 2021; Konteatis et al. 2021). The mechanism involves mRNA splicing dysregulation, R-loop formation, and DNA damage induced by PRMT5 dysfunction, ultimately driving cancer cell death with minimal impact on normal cells, constituting a precise “synthetic lethality” therapeutic strategy (Kalev et al. 2021). Preclinical studies in xenograft models with MTAP deficiency (e.g., the KP4 pancreatic cancer model) validated this strategy, demonstrating that AG-270 dose-dependently reduces intratumoral SAM levels and inhibits tumor growth. Approximately 67% tumor growth inhibition was achieved when SAM levels were reduced by 60–80%, with favorable tolerability (Konteatis et al. 2021). Additionally, the study revealed that MAT2A function extends beyond basal metabolism, forming a positive feedback loop with oncogenic factors (e.g., ERG) in specific environments such as prostate cancer. Its inhibition can synergize with existing therapies (e.g., androgen receptor inhibitors, EZH2 inhibitors), further expanding its therapeutic potential (Cacciatore et al. 2024). The success of this translational medicine concept was preliminarily validated in clinical trials: A Phase I clinical trial (AG-270/S095033) showed that the drug exhibited controllable safety, clear target inhibition signals, and preliminary antitumor activity in patients with advanced MTAP-deficient tumors. Among evaluable patients, 2 cases achieved partial response and 7 cases achieved disease stabilization (including 5 cases with progression-free survival ≥ 16 weeks), with a 16-week disease control rate of 17.5%, providing clinical proof of concept for MAT2A as a therapeutic target (Gounder et al. 2025). In conclusion, the existing evidence from fundamental mechanisms, preclinical efficacy to early clinical translation collectively establishes that targeting MAT2A is a highly promising precision therapy strategy for MTAP-deficient cancers.

PRMT5 catalyzes the SAM-mediated protein arginine methylation, playing a crucial role in tumor biology. MTAP deficiency leads to the accumulation of the metabolite MTA, which is a natural inhibitor of PRMT5. Consequently, novel PRMT5 inhibitors demonstrate significant therapeutic potential for cancers with MTAP gene deletion through a unique “synthetic lethality” mechanism. Among these, AMG 193 is a highly promising PRMT5 inhibitor that exhibits potent activity against MTAP-deficient cancer cells, showing strong cytotoxicity at both cellular and animal model levels with minimal effects on normal hematopoietic cells and no hematotoxicity. Its mechanism of action involves inducing DNA damage, G2/M phase arrest, and splicing defects. AMG 193 exhibits synergistic effects with standard chemotherapeutic agents (such as carboplatin and paclitaxel) and targeted drugs (e.g., sotorasib). In ongoing Phase I and II clinical trials, the drug has effectively controlled and alleviated ovarian support cell-stromal cell tumors and pancreatic cancer without causing toxic effects (Belmontes et al. 2025). Additionally, as the first MTA allosteric co-inhibitor, MRTX1719 demonstrates high therapeutic potential with exceptional binding selectivity (> 70-fold) for the PRMT5•MTA complex, inducing tumor regression in preclinical models. Early clinical data indicate its favorable safety profile, overcoming the dose-limiting toxicity associated with conventional PRMT5 inhibitors (Smith et al. 2022; Engstrom et al. 2023). Similarly, AMG 193 demonstrated encouraging efficacy in the first human Phase I clinical trial for patients with advanced MTAP-deficient solid tumors: among 42 evaluable patients, the objective response rate (ORR) reached 21.4%, and the disease control rate (DCR) was 54.8%. In terms of safety, AMG 193 primarily induced mild to moderate adverse events (commonly nausea, fatigue, and vomiting), with a low incidence of treatment-related adverse events (≥ 3 grade) (13.8%), confirming its favorable efficacy and safety profile (Rodon et al. 2024). These two drugs provide an efficient precision treatment strategy for such patients by precisely leveraging the metabolic vulnerability caused by MTAP deficiency (Fig. 4).

#### Hurdles in targeting methionine metabolic vulnerability

Different strategies targeting methionine metabolism face significant challenges in clinical translation. On one hand, in Phase I clinical trials, the MAT2A inhibitor AG-270 triggered dose-limiting hepatotoxicity at higher doses (200 mg, twice daily), with limited objective response rate (5%) as a monotherapy and failure to achieve expected pharmacokinetic exposure at the highest designed test dose. For these reasons, subsequent clinical development of the drug was suspended (Gounder et al. 2025). Additionally, the first human Phase I study of the PRMT5 inhibitor AMG 193 revealed multiple limitations: firstly, reliance on CDKN2A deletion as a surrogate biomarker for patient screening was imprecise, leading to the inclusion of patients with intact MTAP protein expression who were ultimately ineffective; its monotherapy activity was limited (only 9 out of 80 patients achieved response), and most patients discontinued treatment due to disease progression; furthermore, non-hematologic toxicities such as nausea and fatigue frequently resulted in dose interruption or reduction (Rodon et al. 2024). On the other hand, the dietary intervention strategy of methionine restriction, although promising in terms of mechanism, faces fundamental feasibility bottlenecks in clinical application: strict dietary restrictions and poor palatability severely compromise long-term adherence; prolonged intervention may lead to adverse effects such as loss of lean body mass and bone mineral density; and its efficacy may be weakened by biological compensatory mechanisms such as microbial synthesis of methionine and cysteine, as well as tumor cell uptake of methionine from the microenvironment via transporters (e.g., SLC7A5/SLC43A2) (Parkhitko et al. 2025).

## Key bottlenecks and priority directions for the next decade

### Confronting core bottlenecks: transcending ‘metabolic addiction’

Despite the promising therapeutic paradigm targeting the amino acid metabolic axis, its clinical translation has encountered significant setbacks. The path from convincing preclinical theories to clinical success is fraught with complex biological barriers. Current strategies targeting amino acid metabolism face three critical bottlenecks: On one hand, the metabolic plasticity of tumor cells and adaptive resistance constitute core scientific obstacles. Inhibition of a single node (e.g., GLS1 or arginine) triggers compensatory mechanisms such as isozyme upregulation and bypass activation, enabling tumors to maintain metabolic homeostasis. On the other hand, existing models struggle to fully simulate the metabolic heterogeneity and immune interactions within the human tumor microenvironment, rendering many preclinical-effective strategies ineffective in complex human settings. For instance, the Phase III study of IDO inhibitors combined with immunotherapy failed to meet the endpoint, and numerous targeted drugs (e.g., certain MAT2A inhibitors) were discontinued in early clinical trials due to toxicity or insufficient efficacy, reflecting our current incomplete understanding of the complex in vivo effects of drugs. Finally, the lack of precise patient stratification biomarkers directly hinders clinical success. In the clinical trial of the PRMT5 inhibitor AMG 193, reliance on CDKN2A deletion as a surrogate biomarker for MTAP deficiency was imprecise, leading to the inclusion of patients with intact MTAP protein expression who ultimately failed treatment, highlighting the limitations of existing biomarkers in identifying truly advantageous populations.

### Future priority directions: from mechanisms to precision medicine

To overcome the aforementioned bottlenecks, we propose three major synergistic priority directions for the next decade: 1. Rational combination therapy development: (1) Compensation pathway inhibition and multi-target inhibition strategies: By combining drugs that block key compensatory pathways or simultaneously target multiple nodes within the same metabolic network, drug resistance can be overcome. For example, in pancreatic ductal adenocarcinoma cells, the combination of bis (2-sulfonylpropionyl)oxobenzene (BSO) and the glutaminase inhibitor CB-839 effectively blocks compensation resistance mediated by CB-839 treatment, significantly inhibiting tumor progression. In head and neck squamous cell carcinoma, the combination of CB-839 and CPI-613 produces a synergistic “double metabolic blow” effect; in pancreatic cancer, the combination of CB-839 and the ASCT2 transporter inhibitor V-9302 overcomes adaptive resistance mediated by the GCN2-ATF4 pathway. (2) Metabolic vulnerability synthetic lethality strategy: Combining metabolic inhibitors with other therapies to exploit specific weaknesses generated by metabolic interventions. For example, in MTAP-deficient tumors, the MAT2A inhibitor can be combined with a PRMT5 inhibitor to synergistically reduce intracellular SAM levels through a “synthetic lethality” effect, leading to irreversible mRNA splicing dysregulation and DNA damage in tumor cells. Similarly, in non-small cell lung cancer, the combination of the glutaminase inhibitor CB-839 and the cell cycle-dependent kinase 7 inhibitor THZ simultaneously disrupts tumor cells’ utilization of glucose and glutamine, thereby producing a synergistic antitumor effect. (3) Proactive reversal of metabolic-immune synergy: This represents the most promising research direction, with its core principle being the integration of metabolic interventions and immunotherapy to actively reverse specific immunosuppression induced by metabolic reprogramming while pursuing antitumor effects. For instance, the broad-spectrum glutamine antagonist prodrug DRP-104 and methionine restriction therapy have demonstrated significant efficacy in reversing immunosuppression mediated by amino acid metabolic reprogramming. Furthermore, combining these approaches with anti-PD-1 antibodies can substantially enhance antitumor immunity. 2. Deepening spatiotemporal resolution of metabolic-immune dialogue: It is imperative to dynamically analyze at the single-cell/spatial multi-omics level how specific metabolites precisely regulate immune cell differentiation, function, and exhaustion, particularly the discovery and validation of metabolic checkpoints. 3. Building a translational bridge from preclinical to clinical: There is an urgent need to develop preclinical models that better simulate the human tumor microenvironment and immune system (e.g., humanized mice, organoid co-culture systems) and adopt “umbrella” or “platform” clinical trial designs to rapidly validate biomarker-based combination therapies.

## Conclusion

This review systematically elucidates that targeting the metabolic dependence of tumor cells on specific exogenous amino acids (glutamine, arginine, methionine, tryptophan) represents a highly promising anti-tumor strategy. The various approaches discussed in this article—ranging from enzymatic deprivation, transporter inhibition to dietary restriction—can effectively exploit this vulnerability by inducing direct tumor cell death while reversing the immunosuppressive tumor microenvironment, thereby achieving a synergistic “double whammy” effect. Although significant progress has been made in preclinical and clinical trials targeting the amino acid metabolic axis of tumor cells, fundamental challenges remain in this field. The metabolic plasticity of tumor cells can rapidly offset the inhibition of a single target, and metabolic interventions aimed at killing tumors often produce unpredictable “double-edged sword” effects on immune cells, which may be a key mechanism explaining the suboptimal clinical efficacy of some combination therapies. Future breakthroughs will not only depend on the discovery of new targets but also on the development of multi-target combination strategies to overcome adaptive resistance and precisely guide metabolic interventions to beneficial remodeling of the immune microenvironment. Simultaneously, establishing a functional biomarker system that dynamically reflects the metabolic status of tumors and immune responses is crucial for achieving precision medicine and avoiding clinical trial failures. Overall, targeting amino acid metabolism marks a paradigm shift in oncology, transcending the limitations of traditional genetic targets and fully leveraging metabolic vulnerabilities. Through ongoing research and the application of precision medicine, this strategy is expected to become an indispensable component of future anticancer arsenals.

## Data Availability

The data generated and/or analyzed in this study are accessible from the corresponding author upon reasonable request.

## References

[CR32] Altman BJ, Stine ZE, Dang CV (2016) From Krebs to clinic: glutamine metabolism to cancer therapy. Nat Rev Cancer 16(10):619–634. 10.1038/nrc.2016.71 27492215 10.1038/nrc.2016.71 PMC5484415

[CR51] Beddowes E, Spicer J, Chan PY, Khadeir R, Corbacho JG, Repana D, Steele JP, Schmid P, Szyszko T, Cook G, Diaz M, Feng X, Johnston A, Thomson J, Sheaff M, Wu BW, Bomalaski J, Pacey S, Szlosarek PW (2017) Phase 1 dose-escalation study of pegylated arginine deiminase, cisplatin, and pemetrexed in patients with argininosuccinate synthetase 1-deficient thoracic cancers. J Clin Oncol 35(16):1778–1785. 10.1200/JCO.2016.71.3230 28388291 10.1200/JCO.2016.71.3230 PMC6141244

[CR89] Belmontes B, Slemmons KK, Su C, Liu S, Policheni AN, Moriguchi J, Tan H, Xie F, Aiello DA, Yang Y, Lazaro R, Aeffner F, Rees MG, Ronan MM, Roth JA, Vestergaard M, Cowland S, Andersson J, Sarvary I, Hughes PE (2025) AMG 193, a clinical stage MTA-cooperative PRMT5 inhibitor, drives antitumor activity preclinically and in patients with MTAP-deleted cancers. Cancer Discov 15(1):139–161. 10.1158/2159-8290.CD-24-0887 39282709 10.1158/2159-8290.CD-24-0887 PMC11726016

[CR25] Bian Y, Li W, Kremer DM, Sajjakulnukit P, Li S, Crespo J, Nwosu ZC, Zhang L, Czerwonka A, Pawłowska A, Xia H, Li J, Liao P, Yu J, Vatan L, Szeliga W, Wei S, Grove S, Liu JR, McLean K, Cieslik M, Chinnaiyan AM, Zgodziński W, Wallner G, Wertel I, Okła K, Kryczek I, Lyssiotis CA, Zou W (2020) Cancer SLC43A2 alters T cell methionine metabolism and histone methylation. Nature 585(7824):277–282. 10.1038/s41586-020-2682-1 32879489 10.1038/s41586-020-2682-1 PMC7486248

[CR48] Biancur DE, Paulo JA, Małachowska B, Quiles Del Rey M, Sousa CM, Wang X, Sohn ASW, Chu GC, Gygi SP, Harper JW, Fendler W, Mancias JD, Kimmelman AC (2017) Compensatory metabolic networks in pancreatic cancers upon perturbation of glutamine metabolism. Nat Commun 8:15965. 10.1038/ncomms15965 28671190 10.1038/ncomms15965 PMC5500878

[CR24] Bin P, Wang C, Zhang H, Yan Y, Ren W (2024) Targeting methionine metabolism in cancer: opportunities and challenges. Trends Pharmacol Sci 45(5):395–405. 10.1016/j.tips.2024.03.002 38580603 10.1016/j.tips.2024.03.002

[CR28] Bröer S (2018) Amino acid transporters as disease modifiers and drug targets. SLAS Discov 23(4):303–320. 10.1177/2472555218755629 29557284 10.1177/2472555218755629

[CR87] Cacciatore A, Shinde D, Musumeci C, Sandrini G, Guarrera L, Albino D, Civenni G, Storelli E, Mosole S, Federici E, Fusina A, Iozzo M, Rinaldi A, Pecoraro M, Geiger R, Bolis M, Catapano CV, Carbone GM (2024) Epigenome-wide impact of MAT2A sustains ​the androgen-indifferent state and confers synthetic vulnerability in ERG fusion-positive prostate cancer. Nat Commun 15(1):6672. 10.1038/s41467-024-50908-7 39107274 10.1038/s41467-024-50908-7 PMC11303763

[CR76] Campesato LF, Budhu S, Tchaicha J, Weng CH, Gigoux M, Cohen IJ, Redmond D, Mangarin L, Pourpe S, Liu C, Zappasodi R, Zamarin D, Cavanaugh J, Castro AC, Manfredi MG, McGovern K, Merghoub T, Wolchok JD (2020) Blockade of the AHR restricts a Treg-macrophage suppressive axis induced by L-Kynurenine. Nat Commun 11(1):4011. 10.1038/s41467-020-17750-z 32782249 10.1038/s41467-020-17750-z PMC7419300

[CR17] Cancer Genome Atlas Research Network (2014) Comprehensive molecular profiling of lung adenocarcinoma. Nature 511(7511):543–550. 10.1038/nature13385 25079552 10.1038/nature13385 PMC4231481

[CR14] Chen X, Fu H, Zhu S, Xiang Z, Fu H, Sun Z, Zhang S, Zheng X, Hu X, Chao M, Mao Z, Bi Y, Wang W, Ding Y (2025) The moonlighting function of glutaminase 2 promotes immune evasion of pancreatic ductal adenocarcinoma by tubulin tyrosine ligase-like 1-mediated Yes1 associated transcriptional regulator glutamylation. Gastroenterology 168(6):1137–1152. 10.1053/j.gastro.2025.01.240 39924055 10.1053/j.gastro.2025.01.240

[CR42] Cheng ZJ, Miao DL, Su QY, Tang XL, Wang XL, Deng LB, Shi HD, Xin HB (2019) THZ1 suppresses human non-small-cell lung cancer cells in vitro through interference with cancer metabolism. Acta Pharmacol Sin 40(6):814–822. 10.1038/s41401-018-0187-3 30446732 10.1038/s41401-018-0187-3 PMC6786356

[CR56] De Santo C, Cheng P, Beggs A, Egan S, Bessudo A, Mussai F (2018) Metabolic therapy with PEG-arginase induces a sustained complete remission in immunotherapy-resistant melanoma. J Hematol Oncol 11(1):68. 10.1186/s13045-018-0612-6 29776373 10.1186/s13045-018-0612-6 PMC5960181

[CR6] Dillon BJ, Prieto VG, Curley SA, Ensor CM, Holtsberg FW, Bomalaski JS, Clark MA (2004) Incidence and distribution of argininosuccinate synthetase deficiency in human cancers: a method for identifying cancers sensitive to arginine deprivation. Cancer 100(4):826–833. 10.1002/cncr.20057 14770441 10.1002/cncr.20057

[CR34] Ducker GS, Rabinowitz JD (2017) One-carbon metabolism in health and disease. Cell Metab 25(1):27–42. 10.1016/j.cmet.2016.08.009 27641100 10.1016/j.cmet.2016.08.009 PMC5353360

[CR31] Edwards DN, Ngwa VM, Raybuck AL, Wang S, Hwang Y, Kim LC, Cho SH, Paik Y, Wang Q, Zhang S, Manning HC, Rathmell JC, Cook RS, Boothby MR, Chen J (2021)​​ Selective glutamine metabolism inhibition in tumor cells improves antitumor T lymphocyte activity in triple-negative breast cancer. J Clin Invest 131 (4):e140100. 10.1172/JCI140100 10.1172/JCI140100 PMC7880417 33320840

[CR91] Engstrom LD, Aranda R, Waters L, Moya K, Bowcut V, Vegar L, Trinh D, Hebbert A, Smith CR, Kulyk S, Lawson JD, He L, Hover LD, Fernandez-Banet J, Hallin J, Vanderpool D, Briere DM, Blaj A, Marx MA, Rodon J, Offin M, Arbour KC, Johnson ML, Kwiatkowski DJ, Jänne PA, Haddox CL, Papadopoulos KP, Henry JT, Leventakos K, Christensen JG, Shazer R, Olson P (2023) MRTX1719 is an MTA-cooperative PRMT5 inhibitor that exhibits synthetic l​ethality in preclinical models and patients with MTAP-deleted cancer. Cancer Discov 13:2412–2431. 10.1158/2159-8290.CD-23-0669 37552839 10.1158/2159-8290.CD-23-0669 PMC10618744

[CR62] Enomoto K, Sato F, Tamagawa S, Gunduz M, Onoda N, Uchino S, Muragaki Y, Hotomi M (2019) A novel therapeutic approach for anaplastic thyroid cancer through inhibition of LAT1. Sci Rep 9(1):14616. 10.1038/s41598-019-51144-6 31601917 10.1038/s41598-019-51144-6 PMC6787004

[CR79] Fang L, Hao Y, Yu H, Gu X, Peng Q, Zhuo H, Li Y, Liu Z, Wang J, Chen Y, Zhang J, Tian H, Gao Y, Gao R, Teng H, Shan Z, Zhu J, Li Z, Liu Y, Zhang Y, Yu F, Lin Z, Hao Y, Ge X, Yuan J, Hu HG, Ma Y, Qin HL, Wang P (2023) Methionine restriction promotes cGAS activation and chromatin untethering through demethylation to enhance antitumor immunity. Cancer Cell 41(6):1118–1133e12. 10.1016/j.ccell.2023.05.005 37267951 10.1016/j.ccell.2023.05.005

[CR23] Fujiwara Y, Kato S, Nesline MK, Conroy JM, DePietro P, Pabla S, Kurzrock R (2022) Indoleamine 2,3-dioxygenase (IDO) inhibitors and cancer immunotherapy. Cancer Treat Rev 110:102461. 10.1016/j.ctrv.2022.102461 36058143 10.1016/j.ctrv.2022.102461 PMC12187009

[CR57] Fung MKL, Chan S, Sun S, De Zhang P, Leung GKK, Chan GCF (2025) The therapeutic potential of pegylated arginase I treatment in glioblastoma. Sci Rep 15(1):28994. 10.1038/s41598-025-13882-8 40774999 10.1038/s41598-025-13882-8 PMC12331973

[CR16] Gai X, Liu Y, Lan X, Chen L, Yuan T, Xu J, Li Y, Zheng Y, Yan Y, Yang L, Fu Y, Tang S, Cao S, Dai X, Zhu H, Geng M, Ding J, Pu C, Huang M (2024) Oncogenic KRAS induces arginine auxotrophy and confers a therapeutic vulnerability to SLC7A1 inhibition in non-small cell lung cancer. Cancer Res 84(12):1963–1977. 10.1158/0008-5472.CAN-23-2095 38502865 10.1158/0008-5472.CAN-23-2095

[CR10] Gao P, Tchernyshyov I, Chang TC, Lee YS, Kita K, Ochi T, Zeller KI, De Marzo AM, Van Eyk JE, Mendell JT, Dang CV (2009) c-Myc suppression of miR-23a/b enhances mitochondrial glutaminase expression and glutamine metabolism. Nature 458(7239):762–765. 10.1038/nature07823 19219026 10.1038/nature07823 PMC2729443

[CR81] Gao X, Sanderson SM, Dai Z, Reid MA, Cooper DE, Lu M, Richie JP Jr, Ciccarella A, Calcagnotto A, Mikhael PG, Mentch SJ, Liu J, Ables G, Kirsch DG, Hsu DS, Nichenametla SN, Locasale JW (2019) Dietary methionine influences therapy in mouse cancer models and alters human metabolism. Nature 572(7769):397–401. 10.1038/s41586-019-1437-3 31367041 10.1038/s41586-019-1437-3 PMC6951023

[CR88] Gounder M, Johnson M, Heist RS, Shapiro GI, Postel-Vinay S, Wilson FH, Garralda E, Wulf G, Almon C, Nabhan S, Aguado-Fraile E, He P, Romagnoli M, Hossain M, Narayanaswamy R, Sadou-Dubourgnoux A, Cooper M, Askoxylakis V, Burris HA, Tabernero J (2025) MAT2A inhibitor AG-270/S095033 in patients with advanced malignancies: a phase I trial. Nat Commun 16(1):423. 10.1038/s41467-024-55316-5 39762248 10.1038/s41467-024-55316-5 PMC11704051

[CR13] Gu J, Li Y, Chen Q, Song Z, Qian Q, Liang Y, Huang T, Qiao L, Li X, Yu M, Liu M, Zhou J, Shao Q, Xu X, Zeiser R, Lu L (2026) Tumor-produced ammonia is metabolized by regulatory T cells to further impede anti-tumor immunity. Cell 189(2):418–434. 10.1016/j.cell.2025.11.034 41448182 10.1016/j.cell.2025.11.034

[CR3] Guo J, Satoh K, Tabata S, Mori M, Tomita M, Soga T (2021) Reprogramming of glutamine metabolism via glutamine synthetase silencing induces cisplatin resistance in A2780 ovarian cancer cells. BMC Cancer 21(1):174. 10.1186/s12885-021-07879-5 33596851 10.1186/s12885-021-07879-5 PMC7891143

[CR12] Guo C, You Z, Shi H, Sun Y, Du X, Palacios G, Guy C, Yuan S, Chapman NM, Lim SA, Sun X, Saravia J, Rankin S, Dhungana Y, Chi H (2023) SLC38A2 and glutamine signalling in cDC1s dictate anti-tumour immunity. Nature 620(7972):200–208. 10.1038/s41586-023-06299-8 37407815 10.1038/s41586-023-06299-8 PMC10396969

[CR63] Häfliger P, Graff J, Rubin M, Stooss A, Dettmer MS, Altmann KH, Gertsch J, Charles RP (2018) The LAT1 inhibitor JPH203 reduces growth of thyroid carcinoma in a fully immunocompetent mouse model. J Exp Clin Cancer Res 37(1):234. 10.1186/s13046-018-0907-z 30241549 10.1186/s13046-018-0907-z PMC6150977

[CR58] Hanssen KM, Murray J, Pandher R, Alfred S, Gamble LD, Brand J, Mosmann E, Kusuma FK, Mak C, Kearns A, Kamili A, Atkinson C, Minchaca AZ, Bertoldo J, Ziegler DS, Mussai F, Cheng PNM, Norris MD, Fletcher JI, Haber M (2025) Arginine depletion potentiates standard-of-care chemo-immunotherapy in preclinical models of high-risk neuroblastoma. J Exp Clin Cancer Res 44(1):239. 10.1186/s13046-025-03502-8 40813699 10.1186/s13046-025-03502-8 PMC12351974

[CR2] Huang HY, Wu WR, Wang YH, Wang JW, Fang FM, Tsai JW, Li SH, Hung HC, Yu SC, Lan J, Shiue YL, Hsing CH, Chen LT, Li CF (2013) ASS1 as a novel tumor suppressor gene in myxofibrosarcomas: aberrant loss via epigenetic DNA methylation confers aggressive phenotypes, negative prognostic impact, and therapeutic relevance. Clin Cancer Res 19(11):2861–2872. 10.1158/1078-0432.CCR-12-2641 23549872 10.1158/1078-0432.CCR-12-2641

[CR8] Jain M, Nilsson R, Sharma S, Madhusudhan N, Kitami T, Souza AL, Kafri R, Kirschner MW, Clish CB, Mootha VK (2012) Metabolite profiling identifies a key role for glycine in rapid cancer cell proliferation. Science 336(6084):1040–1044. 10.1126/science.1218595 22628656 10.1126/science.1218595 PMC3526189

[CR83] Jiménez-Alonso JJ, Guillén-Mancina E, Calderón-Montaño JM, Jiménez-González V, Díaz-Ortega P, Burgos-Morón E, López-Lázaro M (2023) Artificial diets with altered levels of sulfur amino acids induce anticancer activity in mice with metastatic colon cancer, ovarian cancer and renal cell carcinoma. Int J Mol Sci 24(5):4587. 10.3390/ijms24054587 36902018 10.3390/ijms24054587 PMC10003419

[CR44] Jin L, Chun J, Pan C, Kumar A, Zhang G, Ha Y, Li D, Alesi GN, Kang Y, Zhou L, Yu WM, Magliocca KR, Khuri FR, Qu CK, Metallo C, Owonikoko TK, Kang S (2018) The PLAG1-GDH1 axis promotes anoikis resistance and tumor metastasis through CamKK2-AMPK signaling in LKB1-deficient lung cancer. Mol Cell 69(1):87–99e7. 10.1016/j.molcel.2017.11.025 29249655 10.1016/j.molcel.2017.11.025 PMC5777230

[CR72] Johnson TS, MacDonald TJ, Pacholczyk R, Aguilera D, Al-Basheer A, Bajaj M, Bandopadhayay P, Berrong Z, Bouffet E, Castellino RC, Dorris K, Eaton BR, Esiashvili N, Fangusaro JR, Foreman N, Fridlyand D, Giller C, Heger IM, Huang C, Kadom N, Kennedy EP, Manoharan N, Martin W, McDonough C, Parker RS, Ramaswamy V, Ring E, Rojiani A, Sadek RF, Satpathy S, Schniederjan M, Smith A, Smith C, Thomas BE, Vaizer R, Yeo KK, Bhasin MK, Munn DH (2024) Indoximod-based chemo-immunotherapy for pediatric brain tumors: a first-in-children phase I trial. Neuro Oncol 26(2):348–361. 10.1093/neuonc/noad174 37715730 10.1093/neuonc/noad174 PMC10836763

[CR69] Jung KH, LoRusso P, Burris H, Gordon M, Bang YJ, Hellmann MD, Cervantes A, Ochoa de Olza M, Marabelle A, Hodi FS, Ahn MJ, Emens LA, Barlesi F, Hamid O, Calvo E, McDermott D, Soliman H, Rhee I, Lin R, Pourmohamad T, Suchomel J, Tsuhako A, Morrissey K, Mahrus S, Morley R, Pirzkall A, Davis SL (2019) Phase I study of the indoleamine 2,3-dioxygenase 1 (IDO1) inhibitor navoximod (GDC-0919) administered with PD-L1 inhibitor (atezolizumab) in advanced solid tumors. Clin Cancer Res 25(11):3220–3228. 10.1158/1078-0432.CCR-18-2740 30770348 10.1158/1078-0432.CCR-18-2740 PMC7980952

[CR84] Kalev P, Hyer ML, Gross S, Konteatis Z, Chen CC, Fletcher M, Lein M, Aguado-Fraile E, Frank V, Barnett A, Mandley E, Goldford J, Chen Y, Sellers K, Hayes S, Lizotte K, Quang P, Tuncay Y, Clasquin M et al (2021) MAT2A inhibition blocks the growth of MTAP-deleted cancer cells by reducing PRMT5-dependent mRNA splicing and inducing DNA damage. Cancer Cell 39(2):209–224e11. 10.1016/j.ccell.2020.12.010 33450196 10.1016/j.ccell.2020.12.010

[CR70] Kelly CM, Qin LX, Whiting KA, Richards AL, Avutu V, Chan JE, Chi P, Dickson MA, Gounder MM, Keohan ML, Movva S, Nacev BA, Rosenbaum E, Adamson T, Singer S, Bartlett EK, Crago AM, Yoon SS, Hwang S, Erinjeri JP, Antonescu CR, Tap WD, D’Angelo SP (2023) A phase II study of epacadostat and pembrolizumab in patients with advanced sarcoma. Clin Cancer Res 29(11):2043–2051. 10.1158/1078-0432.CCR-22-3911 36971773 10.1158/1078-0432.CCR-22-3911 PMC10752758

[CR52] Kim SS, Xu S, Cui J, Poddar S, Le TM, Hayrapetyan H, Li L, Wu N, Moore AM, Zhou L, Yu AC, Dann AM, Elliott IA, Abt ER, Kim W, Dawson DW, Radu CG, Donahue TR (2020) Histone deacetylase inhibition is synthetically lethal with arginine deprivation in pancreatic cancers with low argininosuccinate synthetase 1 expression. Theranostics 10(2):829–840. 10.7150/thno.40195 31903153 10.7150/thno.40195 PMC6929997

[CR40] Kim DH, Kim DJ, Park SJ, Jang WJ, Jeong CH (2025) Inhibition of GLS1 and ASCT2 synergistically enhances the anticancer effects in pancreatic cancer cells. J Microbiol Biotechnol 35:e2412032. 10.4014/jmb.2412.12032 40223274 10.4014/jmb.2412.12032 PMC12010092

[CR85] Konteatis Z, Travins J, Gross S, Marjon K, Barnett A, Mandley E, Nicolay B, Nagaraja R, Chen Y, Sun Y, Liu Z, Yu J, Ye Z, Jiang F, Wei W, Fang C, Gao Y, Kalev P, Hyer ML et al (2021) Discovery of AG-270, a first-in-class oral MAT2A inhibitor for the treatment of tumors with homozygous MTAP deletion. J Med Chem 64(8):4430–4449. 10.1021/acs.jmedchem.0c01895 33829783 10.1021/acs.jmedchem.0c01895

[CR60] Lam SK, Li YY, Xu S, Leung LL, U KP, Zheng YF, Cheng PN, Ho JC (2017) Growth suppressive effect of pegylated arginase in malignant pleural mesothelioma xenografts. Respir Res 18(1):80. 10.1186/s12931-017-0564-3 28464918 10.1186/s12931-017-0564-3 PMC5414232

[CR41] Lang L, Wang F, Ding Z, Zhao X, Loveless R, Xie J, Shay C, Qiu P, Ke Y, Saba NF, Teng Y (2021) Blockade of glutamine-dependent cell survival augments antitumor efficacy of CPI-613 in head and neck cancer. J Exp Clin Cancer Res 40(1):393. 10.1186/s13046-021-02207-y 34906193 10.1186/s13046-021-02207-y PMC8670127

[CR47] Leone RD, Zhao L, Englert JM, Sun IM, Oh MH, Sun IH, Arwood ML, Bettencourt IA, Patel CH, Wen J, Tam A, Blosser RL, Prchalova E, Alt J, Rais R, Slusher BS, Powell JD (2019) Glutamine blockade induces divergent metabolic programs to overcome tumor immune evasion. Science 366(6468):1013–1021. 10.1126/science.aav2588 31699883 10.1126/science.aav2588 PMC7023461

[CR86] Li C, Gui G, Zhang L, Qin A, Zhou C, Zha X (2022) Overview of methionine adenosyltransferase 2A (MAT2A) as an anticancer target: structure, function, and inhibitors. J Med Chem 65(14):9531–9547. 10.1021/acs.jmedchem.2c00395 35796517 10.1021/acs.jmedchem.2c00395

[CR80] Li T, Tan YT, Chen YX, Zheng XJ, Wang W, Liao K, Mo HY, Lin J, Yang W, Piao HL, Xu RH, Ju HQ (2023) Methionine deficiency facilitates antitumour immunity by altering m6A methylation of immune checkpoint transcripts. Gut 72(3):501–511. 10.1136/gutjnl-2022-326928 35803704 10.1136/gutjnl-2022-326928 PMC9933173

[CR18] Lin C, Li Z, Zhu X, Zhou W, Lu X, Zheng J, Lin J (2025) Abnormal β-hydroxybutyrylation modification of ARG1 drives reprogramming of arginine metabolism to promote the progression of colorectal cancer. Adv Sci (Weinh 12(38):e02402. 10.1002/advs.202502402 40641413 10.1002/advs.202502402 PMC12520464

[CR30] Liu Q, Hu J, Li X, Gao H, Kong D, Jin M (2025) ​​ Glutamine transporter inhibitor enhances the sensitivity of NSCLC to trametinib through GSDME-dependent pyroptosis. Biochem Pharmacol 233:116796. 10.1016/j.bcp.2025.116796 39923858 10.1016/j.bcp.2025.116796

[CR68] Long GV, Dummer R, Hamid O, Gajewski TF, Caglevic C, Dalle S, Arance A, Carlino MS, Grob JJ, Kim TM, Demidov L, Robert C, Larkin J, Anderson JR, Maleski J, Jones M, Diede SJ, Mitchell TC (2019) Epacadostat plus pembrolizumab versus placebo plus pembrolizumab in patients with unresectable or metastatic melanoma (ECHO-301/KEYNOTE-252): a phase 3, randomised, double-blind study. Lancet Oncol 20(8):1083–1097. 10.1016/S1470-2045(19)30274-8 31221619 10.1016/S1470-2045(19)30274-8

[CR33] Lukey MJ, Greene KS, Erickson JW, Wilson KF, Cerione RA (2016) The oncogenic transcription factor c-Jun regulates glutaminase expression and sensitizes cells to glutaminase-targeted therapy. Nat Commun 7:11321. 10.1038/ncomms11321 27089238 10.1038/ncomms11321 PMC4837472

[CR77] Martinez Y, Li X, Liu G, Bin P, Yan W, Más D, Valdivié M, Hu CA, Ren W, Yin Y (2017) The role of methionine on metabolism, oxidative stress, and diseases. Amino Acids 49(12):2091–2098. 10.1007/s00726-017-2494-2 28929442 10.1007/s00726-017-2494-2

[CR50] Méndez-Lucas A, Lin W, Driscoll PC, Legrave N, Novellasdemunt L, Xie C, Charles M, Wilson Z, Jones NP, Rayport S, Rodríguez-Justo M, Li V, MacRae JI, Hay N, Chen X, Yuneva M (2020) Identifying strategies to target the metabolic flexibility of tumours. Nat Metab 2(4):335–350. 10.1038/s42255-020-0195-8 32694609 10.1038/s42255-020-0195-8 PMC7436715

[CR43] Miyamoto R, Takigawa H, Yuge R, Shimizu D, Ariyoshi M, Otani R, Tsuboi A, Tanaka H, Yamashita K, Hiyama Y, Urabe Y, Ishikawa A, Sentani K, Oka S (2024) Analysis of anti-tumor effect and mechanism of GLS1 inhibitor CB-839 in colorectal cancer using a stroma-abundant tumor model. Exp Mol Pathol 137:104896. 10.1016/j.yexmp.2024.104896 38703552 10.1016/j.yexmp.2024.104896

[CR9] Muir A, Danai LV, Gui DY, Waingarten CY, Lewis CA, Vander Heiden MG (2017) Environmental cystine drives glutamine anaplerosis and sensitizes cancer cells to glutaminase inhibition. Elife 6:e27713. 10.7554/elife.27713 28826492 10.7554/eLife.27713 PMC5589418

[CR21] Munn DH, Mellor AL (2007) Indoleamine 2,3-dioxygenase and tumor-induced tolerance. J Clin Invest 117(5):1147–1154. 10.1172/JCI31178 17476344 10.1172/JCI31178 PMC1857253

[CR61] Mussai F, Egan S, Higginbotham-Jones J, Perry T, Beggs A, Odintsova E, Loke J, Pratt G, U KP, Lo A, Ng M, Kearns P, Cheng P, De Santo C (2015) Arginine dependence of acute myeloid leukemia blast proliferation: a novel therapeutic target. Blood 125(15):2386–2396. 10.1182/blood-2014-09-600643 25710880 10.1182/blood-2014-09-600643 PMC4416943

[CR39] Okabe S, Tanaka Y, Moriyama M, Gotoh A (2024) Inhibition of glutaminolysis alone and in combination with HDAC inhibitor has anti-myeloma therapeutic effects. Cancer Drug Resist 7:25. 10.20517/cdr.2024.35 39050886 10.20517/cdr.2024.35 PMC11267151

[CR22] Opitz CA, Litzenburger UM, Sahm F, Ott M, Tritschler I, Trump S, Schumacher T, Jestaedt L, Schrenk D, Weller M, Jugold M, Guillemin GJ, Miller CL, Lutz C, Radlwimmer B, Lehmann I, von Deimling A, Wick W, Platten M (2011) An endogenous tumour-promoting ligand of the human aryl hydrocarbon receptor. Nature 478(7368):197–203. 10.1038/nature10491 21976023 10.1038/nature10491

[CR26] Pandit M, Kil YS, Ahn JH, Pokhrel RH, Gu Y, Mishra S, Han Y, Ouh YT, Kang B, Jeong MS, Kim JO, Nam JW, Ko HJ, Chang JH (2023) Methionine consumption by cancer cells drives a progressive upregulation of PD-1 expression in CD4 T cells. Nat Commun 14(1):2593. 10.1038/s41467-023-38316-9 37147330 10.1038/s41467-023-38316-9 PMC10162977

[CR93] Parkhitko AA, Pathak S, Johnson JE, Mittendorfer B, Steinhauser ML (2025) Methionine restriction and mimetics to ameliorate human aging and disease. Trends Endocrinol Metab. 10.1016/j.tem.2025.09.006 41053925 10.1016/j.tem.2025.09.006 PMC13263109

[CR1] Pavlova NN, Thompson CB (2016) The emerging hallmarks of cancer metabolism. Cell Metab 23(1):27–47. 10.1016/j.cmet.2015.12.006 26771115 10.1016/j.cmet.2015.12.006 PMC4715268

[CR59] Prudner BC, Sun F, Kremer JC, Xu J, Huang C, Sai KKS, Morgan Z, Leeds H, McConathy J, Van Tine BA (2018) Amino acid uptake measured by [18F]AFETP increases in response to arginine starvation in ASS1-deficient sarcomas. Theranostics 8(8):2107–2116. 10.7150/thno.22083 29721066 10.7150/thno.22083 PMC5928874

[CR74] Qu X, Wang Y, Jiang Q, Ren T, Guo C, Hua K, Qiu J (2023) Interactions of Indoleamine 2,3-dioxygenase-expressing LAMP3 + dendritic cells with CD4 + regulatory T cells and CD8 + exhausted T cells: synergistically remodeling of the immunosuppressive microenvironment in cervical cancer and therapeutic implications. Cancer Commun (Lond) 43(11):1207–1228. 10.1002/cac2.12486 37794698 10.1002/cac2.12486 PMC10631485

[CR45] Qu T, Song L, Xu J, Lu X, Yin D, Dai J, Zhang C, Guo R, Zhang E (2025) MYLK-AS1 enhances glutamine metabolism to promote EGFR inhibitor resistance in non-small cell lung cancer. Cancer Res 85(16):3052–3071. 10.1158/0008-5472.CAN-23-3748 40366631 10.1158/0008-5472.CAN-23-3748

[CR49] Rais R, Lemberg KM, Tenora L, Arwood ML, Pal A, Alt J, Wu Y, Lam J, Aguilar JMH, Zhao L, Peters DE, Tallon C, Pandey R, Thomas AG, Dash RP, Seiwert T, Majer P, Leone RD, Powell JD, Slusher BS (2022) Discovery of DRP-104, a tumor-targeted metabolic inhibitor prodrug. Sci Adv 8(46):eabq5925. 10.1126/sciadv.abq5925 36383674 10.1126/sciadv.abq5925 PMC9668306

[CR38] Raninga PV, He Y, Datta KK, Lu X, Maheshwari UR, Venkat P, Mayoh C, Gowda H, Kalimutho M, Hooper JD, Khanna KK (2023) Combined thioredoxin reductase and glutaminase inhibition exerts synergistic anti-tumor activity in MYC-high high-grade serous ovarian carcinoma. Mol Ther 31(3):729–743. 10.1016/j.ymthe.2022.12.011 36560881 10.1016/j.ymthe.2022.12.011 PMC10014232

[CR64] Rii J, Sakamoto S, Mizokami A, Xu M, Fujimoto A, Saito S, Koike H, Tamura T, Arai T, Yamada Y, Goto Y, Sazuka T, Imamura Y, Suzuki K, Kanai Y, Anzai N, Ichikawa T (2024) L-type amino acid transporter 1 inhibitor JPH203 prevents the growth of cabazitaxel-resistant prostate cancer by inhibiting cyclin-dependent kinase activity. Cancer Sci 115(3):937–953. 10.1111/cas.16062 38186218 10.1111/cas.16062 PMC10920979

[CR92] Rodon J, Prenen H, Sacher A, Villalona-Calero M, Penel N, El Helali A, Rottey S, Yamamoto N, Ghiringhelli F, Goebeler ME, Doi T, Postel-Vinay S, Lin CC, Liu C, Chuang CH, Keyvanjah K, Eggert T, O’Neil BH (2024) First-in-human study of AMG 193, an MTA-cooperative PRMT5 inhibitor, in patients with MTAP-deleted solid tumors: results from phase I dose exploration. Ann Oncol 35:1138–1147. 10.1016/j.annonc.2024.08.2339 39293516 10.1016/j.annonc.2024.08.2339

[CR19] Rodriguez PC, Ochoa AC, Al-Khami AA (2017) Arginine metabolism in myeloid cells shapes innate and adaptive immunity. Front Immunol 8:93. 10.3389/fimmu.2017.00093 28223985 10.3389/fimmu.2017.00093 PMC5293781

[CR7] Rogers LC, Zhou J, Baker A, Schutt CR, Panda PK, Van Tine BA (2021) Intracellular arginine-dependent translation sensor reveals the dynamics of arginine starvation response and resistance in ASS1-negative cells. Cancer Metab 9(1):4. 10.1186/s40170-021-00238-9 33478587 10.1186/s40170-021-00238-9 PMC7818940

[CR27] Schulte ML, Fu A, Zhao P, Li J, Geng L, Smith ST, Kondo J, Coffey RJ, Johnson MO, Rathmell JC, Sharick JT, Skala MC, Smith JA, Berlin J, Washington MK, Nickels ML, Manning HC (2018)​​ Pharmacological blockade of ASCT2-dependent glutamine transport leads to antitumor efficacy in preclinical models. Nat Med 24 (2):194–202. 10.1038/nm.4464 10.1038/nm.4464 PMC5803339 29334372

[CR90] Smith CR, Aranda R, Bobinski TP, Briere DM, Burns AC, Christensen JG, Clarine J, Engstrom LD, Gunn RJ, Ivetac A, Jean-Baptiste R, Ketcham JM, Kobayashi M, Kuehler J, Kulyk S, Lawson JD, Moya K, Olson P, Rahbaek L, Thomas NC, Wang X, Waters LM, Marx MA (2022) Fragment-based discovery of MRTX1719, a synthetic lethal inhibitor of the PRMT5•MTA complex for the treatment of MTAP-deleted cancers. J Med Chem 65:1749–1766. 10.1021/acs.jmedchem.1c01900 35041419 10.1021/acs.jmedchem.1c01900

[CR11] Son J, Lyssiotis CA, Ying H, Wang X, Hua S, Ligorio M, Perera RM, Ferrone CR, Mullarky E, Shyh-Chang N, Kang Y, Fleming JB, Bardeesy N, Asara JM, Haigis MC, DePinho RA, Cantley LC, Kimmelman AC (2013) Glutamine supports pancreatic cancer growth through a KRAS-regulated metabolic pathway. Nature 496(7443):101–105. 10.1038/nature12040 23535601 10.1038/nature12040 PMC3656466

[CR53] Szlosarek PW, Creelan BC, Sarkodie T, Nolan L, Taylor P, Olevsky O, Grosso F, Cortinovis D, Chitnis M, Roy A, Gilligan D, Kindler H, Papadatos-Pastos D, Ceresoli GL, Mansfield AS, Tsao A, O’Byrne KJ, Nowak AK, Steele J, Sheaff M, Shiu CF, Kuo CL, Johnston A, Bomalaski J, Zauderer MG, Fennell DA, ATOMIC-Meso Study Group (2024) Pegargiminase Plus First-Line Chemotherapy in Patients With Nonepithelioid Pleural Mesothelioma: The ATOMIC-Meso Randomized Clinical Trial. JAMA Oncol 10(4):475–483. 10.1001/jamaoncol.2023.6789 38358753 10.1001/jamaoncol.2023.6789 PMC10870227

[CR36] Tambay V, Raymond VA, Turcotte S, Bilodeau M (2025) Glutaminase Reprogramming in Hepatocellular Carcinoma: Implications for Diagnosis, Prognosis, and Potential as a Novel Therapeutic Target. Int J Mol Sci 26(19):9653. 10.3390/ijms26199653 41096916 10.3390/ijms26199653 PMC12524534

[CR71] Tărlungeanu DC, Deliu E, Dotter CP, Kara M, Janiesch PC, Scalise M, Galluccio M, Tesulov M, Morelli E, Sonmez FM, Bilguvar K, Ohgaki R, Kanai Y, Johansen A, Esharif S, Ben-Omran T, Topcu M, Schlessinger A, Indiveri C, Duncan KE, Caglayan AO, Gunel M, Gleeson JG, Novarino G (2016) Impaired Amino Acid Transport at the Blood Brain Barrier Is a Cause of Autism Spectrum Disorder. Cell 167(6):1481–1494e18. 10.1016/j.cell.2016.11.013 27912058 10.1016/j.cell.2016.11.013 PMC5554935

[CR75] Triplett TA, Garrison KC, Marshall N, Donkor M, Blazeck J, Lamb C, Qerqez A, Dekker JD, Tanno Y, Lu WC, Karamitros CS, Ford K, Tan B, Zhang XM, McGovern K, Coma S, Kumada Y, Yamany MS, Sentandreu E, Fromm G, Tiziani S, Schreiber TH, Manfredi M, Ehrlich LIR, Stone E, Georgiou G (2018) Reversal of indoleamine 2,3-dioxygenase-mediated cancer immune suppression by systemic kynurenine depletion with a therapeutic enzyme. Nat Biotechnol 36(8):758–764. 10.1038/nbt.4180 30010674 10.1038/nbt.4180 PMC6078800

[CR54] Tsai HJ, Jiang SS, Hung WC, Borthakur G, Lin SF, Pemmaraju N, Jabbour E, Bomalaski JS, Chen YP, Hsiao HH, Wang MC, Kuo CY, Chang H, Yeh SP, Cortes J, Chen LT, Chen TY (2017) A phase II study of arginine deiminase (ADI-PEG20) in relapsed/refractory or poor-risk acute myeloid leukemia patients. Sci Rep 7(1):11253. 10.1038/s41598-017-10542-4 28900115 10.1038/s41598-017-10542-4 PMC5595917

[CR4] Vettore L, Westbrook RL, Tennant DA (2020) New aspects of amino acid metabolism in cancer. Br J Cancer 122(2):150–156. 10.1038/s41416-019-0620-5 31819187 10.1038/s41416-019-0620-5 PMC7052246

[CR5] Wang D, Wan X (2022) Progress in research on the role of amino acid metabolic reprogramming in tumour therapy: a review. Biomed Pharmacother 156:113923. 10.1016/j.biopha.2022.113923 36411616 10.1016/j.biopha.2022.113923

[CR35] Wang Z, Yip LY, Lee JHJ, Wu Z, Chew HY, Chong PKW, Teo CC, Ang HY, Peh KLE, Yuan J, Ma S, Choo LSK, Basri N, Jiang X, Yu Q, Hillmer AM, Lim WT, Lim TKH, Takano A, Tan EH, Tan DSW, Ho YS, Lim B, Tam WL (2019) Methionine is a metabolic dependency of tumor-initiating cells. Nat Med 25(5):825–837. 10.1038/s41591-019-0423-5 31061538 10.1038/s41591-019-0423-5

[CR46] Wang Q, Wu M, Li H, Rao X, Ao L, Wang H, Yao L, Wang X, Hong X, Wang J, Aa J, Sun M, Wang G, Liu J, Zhou F (2022) Therapeutic targeting of glutamate dehydrogenase 1 that links metabolic reprogramming and Snail-mediated epithelial-mesenchymal transition in drug-resistant lung cancer. Pharmacol Res 185:106490. 10.1016/j.phrs.2022.106490 36216131 10.1016/j.phrs.2022.106490

[CR37] Wang H, Wang XY, Ji JB, Zheng ZX, Shang PF, Guo XL (2026) GLS1 inhibitor CB-839 inhibits the malignant progression of 5-FU resistant hepatoma cells by regulating glutamine metabolism. Chem Biol Interact 423:111812. 10.1016/j.cbi.2025.111812 41213394 10.1016/j.cbi.2025.111812

[CR67] Watanabe T, Gaedicke S, Guffart E, Firat E, Niedermann G (2020) Adding indoximod to hypofractionated radiotherapy with anti-PD-1 checkpoint blockade enhances early NK and CD8 + T-cell-dependent tumor activity. Clin Cancer Res 26(4):945–956. 10.1158/1078-0432.CCR-19-0476 31694834 10.1158/1078-0432.CCR-19-0476

[CR78] Wei F, Locasale JW (2023) Methionine restriction and antitumor immunity. Trends Cancer 9(9):705–706. 10.1016/j.trecan.2023.07.008 37517954 10.1016/j.trecan.2023.07.008 PMC10458792

[CR82] Yamamoto J, Miyake K, Han Q, Tan Y, Inubushi S, Sugisawa N, Higuchi T, Tashiro Y, Nishino H, Homma Y, Matsuyama R, Chawla SP, Bouvet M, Singh SR, Endo I, Hoffman RM (2020) )​Oral recombinant methioninase increases TRAIL receptor-2 expression to regress pancreatic cancer in combination with agonist tigatuzumab in an orthotopic mouse model. Cancer Lett 492:174–184. 10.1016/j.canlet.2020.07.034 32739322 10.1016/j.canlet.2020.07.034

[CR15] Yang Y, Pei T, Liu C, Cao M, Hu X, Yuan J, Chen F, Guo B, Hong Y, Liu J, Li B, Li X, Wang H (2025) Glutamine metabolic competition drives immunosuppressive reprogramming of intratumour GPR109A+ myeloid cells to promote liver cancer progression. Gut 74(2):255–269. 10.1136/gutjnl-2024-332429 10.1136/gutjnl-2024-332429 38981667

[CR55] Yao S, Janku F, Subbiah V, Stewart J, Patel SP, Kaseb A, Westin SN, Naing A, Tsimberidou AM, Hong D, Piha-Paul SA, Shi N, Johnston A, Bomalaski J, Fu S (2021) Phase 1 trial of ADI-PEG20 plus cisplatin in patients with pretreated metastatic melanoma or other advanced solid malignancies. Br J Cancer 124(9):1533–1539. 10.1038/s41416-020-01230-8 33674736 10.1038/s41416-020-01230-8 PMC8076217

[CR73] Zakharia Y, McWilliams RR, Rixe O, Drabick J, Shaheen MF, Grossmann KF, Kolhe R, Pacholczyk R, Sadek R, Tennant LL, Smith CM, Kennedy EP, Link CJ Jr, Vahanian NN, Yu J, Shen SS, Brincks EL, Rossi GR, Munn D, Milhem M (2021) Phase II trial of the IDO pathway inhibitor indoximod plus pembrolizumab for the treatment of patients with advanced melanoma. J Immunother Cancer 9(6):e002057. 10.1136/jitc-2020-002057 34117113 10.1136/jitc-2020-002057 PMC8202104

[CR29] Zhang Z, Liu R, Shuai Y, Huang Y, Jin R, Wang X, Luo J (2020) )ASCT2 (SLC1A5)-dependent glutamine uptake is involved in the progression of head and neck squamous cell carcinoma. Br J Cancer 122(1):82–93. 10.1038/s41416-019-0637-9 31819178 10.1038/s41416-019-0637-9 PMC6964701

[CR65] Zhang YW, Velasco-Hernandez T, Mess J, Lalioti ME, Romero-Mulero MC, Obier N, Karantzelis N, Rettkowski J, Schönberger K, Karabacz N, Jäcklein K, Morishima T, Trincado JL, Romecin P, Martinez A, Takizawa H, Shoumariyeh K, Renders S, Zeiser R, Pahl HL, Béliveau F, Hébert J, Lehnertz B, Sauvageau G, Menendez P, Cabezas-Wallscheid N (2023) GPRC5C drives branched-chain amino acid metabolism in leukemogenesis. Blood Adv 7(24):7525–7538. 10.1182/bloodadvances.2023010460 37639313 10.1182/bloodadvances.2023010460 PMC10761356

[CR66] Zhao Y, Pu C, Liu K, Liu Z (2025) Targeting LAT1 with JPH203 to reduce TNBC proliferation and reshape suppressive immune microenvironment by blocking essential amino acid uptake. Amino Acids 57(1):27. 10.1007/s00726-025-03456-3 40379991 10.1007/s00726-025-03456-3 PMC12084285

[CR20] Zhu Y, Zhou Z, Du X, Lin X, Liang ZM, Chen S, Sun Y, Wang Y, Na Z, Wu Z, Zhong J, Han B, Zhu X, Fu W, Li H, Luo ML, Hu H (2025) Cancer cell-derived arginine fuels polyamine biosynthesis in tumor-associated macrophages to promote immune evasion. Cancer Cell 43(6):1045–1060e7. 10.1016/j.ccell.2025.03.015 40185095 10.1016/j.ccell.2025.03.015

